# CO_2_ assimilation strategies in stratified lakes: Diversity and distribution patterns of chemolithoautotrophs

**DOI:** 10.1111/1462-2920.13786

**Published:** 2017-05-29

**Authors:** Albin Alfreider, Andreas Baumer, Teresa Bogensperger, Thomas Posch, Michaela M. Salcher, Monika Summerer

**Affiliations:** 1 Institute for Ecology, University of Innsbruck, Innsbruck, Austria; 2 Limnological Station, Department of Plant and Microbial Biology, University of Zurich, Kilchberg, Switzerland; 3 Institute of Hydrobiology, Biology Centre CAS, České Budějovice, Czech Republic

## Abstract

While mechanisms of different carbon dioxide (CO_2_) assimilation pathways in chemolithoautotrohic prokaryotes are well understood for many isolates under laboratory conditions, the ecological significance of diverse CO_2_ fixation strategies in the environment is mostly unexplored. Six stratified freshwater lakes were chosen to study the distribution and diversity of the Calvin-Benson-Bassham (CBB) cycle, the reductive tricarboxylic acid (rTCA) cycle, and the recently discovered archaeal 3-hydroxypropionate/4-hydroxybutyrate (HP/HB) pathway. Eleven primer sets were used to amplify and sequence genes coding for selected key enzymes in the three pathways. Whereas the CBB pathway with different forms of RubisCO (IA, IC and II) was ubiquitous and related to diverse bacterial taxa, encompassing a wide range of potential physiologies, the rTCA cycle in *Epsilonproteobacteria* and *Chloribi* was exclusively detected in anoxic water layers. Nitrifiying *Nitrosospira* and *Thaumarchaeota*, using the rTCA and HP/HB cycle respectively, are important residents in the aphotic and (micro-)oxic zone of deep lakes. Both taxa were of minor importance in surface waters and in smaller lakes characterized by an anoxic hypolimnion. Overall, this study provides a first insight on how different CO_2_ fixation strategies and chemical gradients in lakes are associated to the distribution of chemoautotrophic prokaryotes with different functional traits.

## Introduction

Concerns about global warming have focussed the attention to biological processes that influence the removal or sequestration of inorganic carbon from aquatic ecosystems. The most important global sink is photosynthetic carbon dioxide fixation (CO_2_) (Field *et al*., 1998). The role of chemolithoautotrophic prokaryotes for reduction of inorganic carbon has received minor attention, presumably because chemolithoautotrophs are considered to be of minor importance in the global gross primary productivity, as reported for marine ecosystems (Middelburg, 2011) and aquatic systems in general (Raven, 2009). Recent research activities challenged this view. For marine systems, several studies suggest that chemolithoautotrophy is not only of significance in hydrothermal vents, but also in the meso- and bathypelagic environment (Hügler and Sievert, 2011; Swan *et al*., 2011; Herndl and Reinthaler, 2013) and in benthic deep-sea sediments (Molari *et al*., 2013). In lacustrine environments and specifically in lakes, the importance of chemolithoautotrophic processes is typically depending on the morphometric and chemical characteristics of the system. Several studies suggest a substantial contribution by chemoautotrophic organisms to the carbon cycle measured by dark CO_2_ fixation rates, particularly in lakes characterized by sulfidic redoxclines (e.g. Casamayor, 2010; Noguerola *et al*., 2015) or in the hypolimnion of deep lakes (Callieri *et al*., 2014).

Currently, six pathways are known by which chemolithoautotrophic organisms fix CO_2_, whereby the Calvin-Benson-Bassham (CBB) cycle, the reductive tricarboxylic acid (rTCA) cycle, the reductive acetyl-CoA pathway and the recently discovered 3-hydroxypropionate/4-hydroxybutyrate (HP/HB) cycle are most widely distributed in different habitats (Berg, 2011). In environmental samples, however, the identification and differentiation of prokaryotes harbouring these pathways is hampered by methodological challenges. Different CO_2_ fixation pathways are often widespread among prokaryotes and usually not related to distinct taxonomic groups (Berg, 2011). Consequently, phylogenetic markers (e.g. based on 16S rRNA) are not sufficient for the identification of CO_2_ fixation strategies and associated energy metabolisms. The analysis of functional genes, coding for key enzymes in the different inorganic carbon assimilation pathways, provides an excellent strategy for the investigation of specific CO_2_ fixation strategies in environmental samples (Hügler and Sievert, 2011).

In this respect, ribulose-1,5-bisphosphate carboxylase/oxygenase (RubisCO) is the most prominent enzyme, and the gene coding for the large subunit of RubisCO is known to serve as a marker for the analysis of autotrophic organisms, including bacteria, using the CBB cycle (Badger and Bek, 2008; Berg, 2011). From the structural viewpoint, two types of RubisCO are known: form I and form II, with 25 to 30% amino acid sequence identity respectively (Tabita *et al*., 2008). Phylogenetic analyses divide form I RubisCO into two groups (‘green’ and ‘red’) which may be further subdivided into types IA, IB, IC, ID (Tabita *et al*., 2008). Several examined bacterial genome sequences indicate that there are even two different types of form IA structures, with form IAc being associated with carboxysomes (Badger and Bek, 2008). The form II RubisCO enzyme, until recently known only from *Proteobacteria*, is markedly different from form I. Important biochemical features of form II enzymes are their poor affinity to CO_2_ and the low specificity factor τ, a measure of the ability of the RubisCO to discriminate between CO_2_ and O_2_ at a given CO_2_:O_2_ concentration ratio. Consequently, form II enzymes are adapted to low-O_2_ and high-CO_2_ environments (Badger and Bek, 2008). Forms IA, IC and II RubisCO phylotypes are mostly found in chemolithoautotrophic bacteria. In freshwater lakes, the diversity and distribution of RubisCO among photoautotrophic organisms is well known (e.g. Xu and Tabita, 1996; Wilhelm *et al*., 2006; Kong *et al*., 2012b). Investigations of RubisCO genes affiliated with chemolithoautotrophic planktonic freshwater bacteria are rare and include lakes characterized by saline and/or alkaline conditions (Giri *et al*., 2004; Tourova *et al*., 2010; Kovaleva *et al*., 2011), an Antarctic lake (Kong *et al*., 2012a), and samples of temperate lakes studied by high-throughput single-cell sequencing (Martinez-Garcia *et al*., 2012).

Beside chemolithoautotrophs using the Calvin cycle, there is currently an increased interest in the exploration of *Thaumarchaeota*, which assimilate inorganic carbon with an energy efficient variant of the HP/HB cycle originally described for hyperthermophilic *Crenarchaeota* (Könneke *et al*., 2014). Genes coding for key enzymes in this CO_2_ fixation pathway were mainly investigated in marine systems (e.g. Yakimov *et al*., 2009; 2011; Bergauer *et al*., 2013; Hu *et al*., 2013; Tolar *et al*., 2013), while there are currently only two studies in freshwater lakes (Auguet *et al*., 2008; La Cono *et al*., 2013). Ammonia is often used as a sole energy source and *amoA* gene surveys suggest that autotrophic *Thaumarchaeota* using the HP/HB pathway for CO_2_ fixation are major players in the nitrogen cycle of aquatic systems (Bouskill *et al*., 2012; Hugoni *et al*., 2013; Vissers *et al*., 2013).

The rTCA cycle operates in phylogenetically diverse autotrophic bacteria including genera of anoxic phototrophic bacteria, sulfate-reducing bacteria, and hyperthermophilic hydrogen-oxidizing bacteria (Hügler *et al*., 2005). Autotrophic *Epsilonproteobacteria* and *Aquificales* generally fix CO_2_ via the rTCA pathway. CO_2_ fixation based on the rTCA pathway was also shown for several bacterial representatives phylogenetically related to *Chlorobiales*, *Deltaproteobacteria*, and magnetotactic cocci affiliated with the class of *Alphaproteobacteria*, but their environmental importance has yet to be determined (Hügler and Sievert, 2011). In addition, (meta)genomic analyses suggest the operation of the rTCA cycle in autotrophic members of nitrite oxidizing and ammonia oxidizing *Nitrospirae* (Lücker *et al*., 2010; Daims *et al*., 2015; 2016). Environmental investigations based on the detection of genes coding for key enzymes in the rTCA pathway were mainly conducted in hydrothermal vent systems (reviewed by Hügler and Sievert, 2011), dominated by autotrophic *Epsilonproteobacteria* and *Aquificales*. For lakes, we are aware of two studies specifically exploring the distribution of the rTCA cycle, which includes the analysis of ATP citrate lyase genes in soda lake sediments (Kovaleva *et al*., 2011) and the sulfidic redoxcline of a meromictic karstic lake (Noguerola *et al*., 2015).

The objective of this study was to explore the distribution and diversity of different chemolithoautotrophic CO_2_ fixation strategies in six stratified lakes. In this respect, stratified lakes are ideal habitats for studying chemolithoautotrophs, because these ecosystems are characterized by distinct concentration gradients of oxygen and different redox states of nitrogen, sulfur, and other elements, thus allowing the investigation of chemolithoautotrophs in the ecological framework of measurable habitat heterogeneity. From preliminary investigations, we anticipated that the CBB cycle, the rTCA cycle, and the HP/HB cycle are of significance in these lakes, with pronounced variations of spatial scales within- and among-lakes. Our study focussed on the analysis of genes coding for selected key enzymes for the assimilation of CO_2_ in these three pathways. Furthermore, *Thaumarchaeota* and the related HP/HB cycle were quantified based on CARD-FISH and qPCR targeting functional genes.

## Results and discussion

### Physico-chemical stratification patterns

**Fig. 1:**
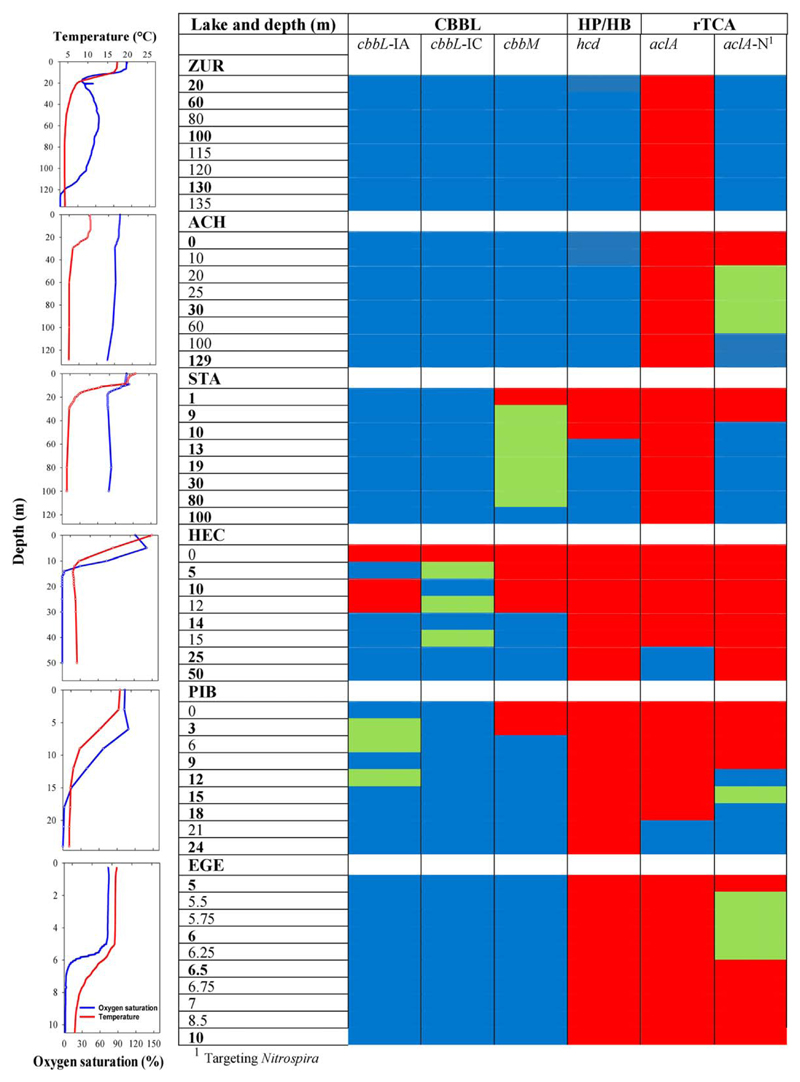
Distribution of CO_2_ fixation mechanisms based on the detection of genes coding for different key enzymes in the individual pathways. A blue colour-code defines a specific amplification product, a green colour indicates multiple bands. Samples designated with a red colour produced no PCR-band. Sampling depths, which were chosen for the construction of clone libraries and sequence analysis, are shown in bold. Lake stratification patterns at the sampling date, specified by depth profiles of oxygen and temperature, are shown on the left margin.

We sampled the depth profiles of six European lakes, whereof three were larger than 6 km^2^ and deeper than 120 m (Lake Zurich – ZUR, Achensee – ACH, Starnberger See – STA), the three others were much smaller than 1 km^2^ and 10.5 to 57 m deep (Hechtsee – HEC, Piburger See – PIB, Egelsee – EGE; Table 1). At the sampling dates, all six lakes were characterized by typical (late) summer stratification patterns of the water column, with pronounced differences in the sharpness of their chemical gradients, the dimension and depth of the hypolimnion, and the spatial distribution of different redox conditions (Fig. 1, Supporting Information Table S2). ZUR showed a distinct metalimnetic oxygen minimum and oxygen concentrations decreased again significantly below 100 m depth. Sampling depths at 130 and 135 m were free of oxygen and rich in ammonium (Supporting Information Fig. S6). Although water temperature in the epilimnion was lower in ACH (sampling date October) than in STA (sampled end of August), oxygen profiles were comparable in both lakes with oxygen-saturations always above 80% in the entire water columns of 129 and 100 m depths respectively. Accordingly, both lakes were characterized by the absence or very low concentrations of reduced compounds and low nutrient levels were characteristic for the oligotrophic nature of both lakes (Supporting Information Table S2). HEC is permanently meromictic, characterized by anoxic conditions from 15 m downward to the deepest sampling depth in 50 m (Fig. 1). The hypolimnion showed high concentrations of phosphorous and ammonium (Supporting Information Table S2 and Fig. S6). Oxygen concentration in dimictic PIB started to decrease at a depth of 6 m and sampling depths between 18 and 24 m were free of oxygen (Supporting Information Fig. S6). Analysis of the water column of EGE revealed a pronounced stratification, characterized by a strong gradient in the water chemistry and an anoxic hypolimnion (Fig. 1; Supporting Information Table S2). Dissolved sulfide was not measured directly, however, the decrease of sulfate, the absence of oxygen and a sulfide odour were observed in samples of the hypolimnion of HEC, EGE and the deepest sampling depth of PIB, indicating sulfidic conditions in these samples. Although sulfide was not measured in large lakes ACH, STA and ZUR, oxygen concentration indicate that all samples, with exception of ZUR 130m/135m, were free of sulfide (Supporting Information Table S2).

### Diversity and distribution of different forms of RubisCO in the CBB cycle

Five broad-range primer pairs were used to investigate the diversity of form IA, form IC and form II types of RubisCO genes, which comprise the majority of RubisCO’s to be found in chemolithoautotrophic bacteria using the CBB cycle for CO_2_ fixation. All three forms of RubisCO were detected in most sampling depths of all six lakes, suggesting that CO_2_ fixation via the Calvin-Benson-Bassham pathway is a major pathway at our study sites and that the CBB cycle is operational under different environmental conditions (Fig. 1). An exception was the epilimnion of HEC, where RubisCO genes were mostly affiliated with cyanobacterial sequences (Supporting Information Fig. S1A). In PIB and STA, *cbbM* genes were absent in the surface waters (Fig. 1).

One third of form IA RubisCO sequences obtained from all six lakes were affiliated with cyanobacterial lineages, which were also covered by the broad range primer used. They comprised the majority of sequences in the epilimnion of the lakes. Taxonomically, two closely related major clades in the cyanobacterial branch were grouped with different *Synechococcus* spp. and *Paulinella chromatophora* chloroplasts. Chemoautotrophs containing form IA RubisCO sequences were mostly related with *Betaproteobacteria* (Supporting Information Fig. S1A). In terms of abundance (i.e. number of sequences) and distribution, other bacterial groups including *Alphaproteobacteria*, *Gammaproteobacteria*, and *Actinobacteria* were of minor importance at our study sites (Supporting Information Fig. S1A). The metabolic lifestyle, derived from physiological properties of the closest cultured relatives, was dominated by obligate and potentially facultative chemoautotrophs, with nitrification as major source of energy. Although RubisCO form IA sequences related to chemolithoautotrophs displayed a broad diversity in the individual lakes and sampling depths, two major clades of closely related sequences stand out noticeably (Supporting Information Fig. S1A). The first clade is composed of 92 sequences, which were obtained from all lakes with exception of EGE. These sequences are affiliated with the obligate chemolithoautotrophic ammonia-oxidizing bacteria *Nitrosomonas* sp. AL212 and *Nitrosomonas* sp. Is79A3, both representatives of the *Nitrosomonas oligotropha* lineage (cluster 6A), and *Nitrosomonas cryotolerans*. Most members of the *N. oligotropha* lineage are known for their ability to grow effectively at low ammonium concentrations (Suwa *et al*., 2011; Bollmann *et al*., 2013). The second clade contains 81 sequences of the smaller lakes EGE, PIB, and HEC, derived from sample depths characterized by microaerobic or anaerobic conditions. This cluster is distantly related to the Gram positive bacterium *Actinoplanes subtropicus*, an organism known for its heterotrophic lifestyle (Seong and Soon, 2009). Based on the limited data and phylogenetic isolation of this clade, the function and taxonomy of bacteria harbouring this RubisCO form IA phylotype is unclear.

*Betaproteobacteria* were also the most important class of bacteria affiliated with RubisCO form IC sequences (Supporting Information Fig. S1B). Other bacterial taxa could not clearly be assigned, but green non-sulfur bacteria of the phylum *Chloroflexi* were likely present in EGE. The form IC RubisCO tree is dominated by one major clade with 266 highly similar sequences, which were detected in all lakes and almost all sampling depths examined (Supporting Information Fig. S1B). Sequences of this cluster were affiliated with different RubisCO genotypes obtained by cultivation independent analysis from aquifer samples (Alfreider *et al*., 2009). The closest cultivated relative is the *Hydrogenophaga* sp. RAC07, isolated from freshwater algae *Chrysochromulina tobin* phycosphere (Fixen *et al*., 2016). Information on the ecological properties of *Hydrogenophaga* sp. RAC07 were not available at the time of publication, but the type of growth medium used for the isolation Hydrogenophaga sp. RAC07 was composed of organic carbon substrates supporting heterotrophic growth (Fixen *et al*., 2016).

RubisCO Form II sequences revealed a high diversity of different groups of *Proteobacteria* (Supporting Information Fig. S1C). *CbbM* sequences clustering with dinoflagellates were retrieved from HEC and ACH. In contrast to *cbbL* sequences obtained in this study, the phylogenetic tree does not contain major clades of highly similar sequences related to one specific metabolism or taxon. One exception is a cluster of sequences obtained from different depth in the hypolimnion of lakes EGE, HEC, PIB, ZUR and the surface water sample of ACH, which was clearly affiliated with *Sulfuricella denitrificans* skB26. This bacterium was recently isolated from a freshwater lake and described as a psychrotolerant sulfur-oxidizing betaproteobacterium (Kojima and Fukui, 2010; Watanabe *et al*., 2012). The phylogenetic affiliation of *cbbM* sequences allows only limited interpretation on the ecophysiology and associated energy metabolisms. Closest relatives include numerous obligate and facultative chemolithoautotrophs, photoheterotrophs and anoxygenic photoautotrophs (Supporting Information Fig. S1C).

Unfortunately, our investigation strategy based on bulk DNA does not reveal whether different forms of RubisCO are present within a single cell or in different bacteria. However, form II RubisCO is often found in organisms that also contain form I (Badger and Bek, 2008), though even three different forms of RubisCO have been detected in a single organism (e.g. Yoshizawa *et al*., 2004). We anticipate that chemoautotrophic microorganisms that have retained the genes encoding forms I and II of RubisCO may play an important role in oxic/anoxic boundaries of lakes, because the different kinetic properties of the enzymes would allow efficient CO_2_ fixation under both aerobic and anoxic conditions.

### Diversity and abundance of *Thaumarchaeota* and functional genes in the HP/HB cycle

Genes coding for 4-hydroxybutyryl-CoA dehydratase (*hcd*) produced specific PCR amplicons in most depths of the deep lakes ACH, STA and ZUR, with exception of the epilimnion of STA (Fig. 1). The same primer pairs did not yield products in the smaller lakes HEC, PIB and EGE, suggesting that the HP/HB pathway is absent at these sites.

**Fig. 2:**
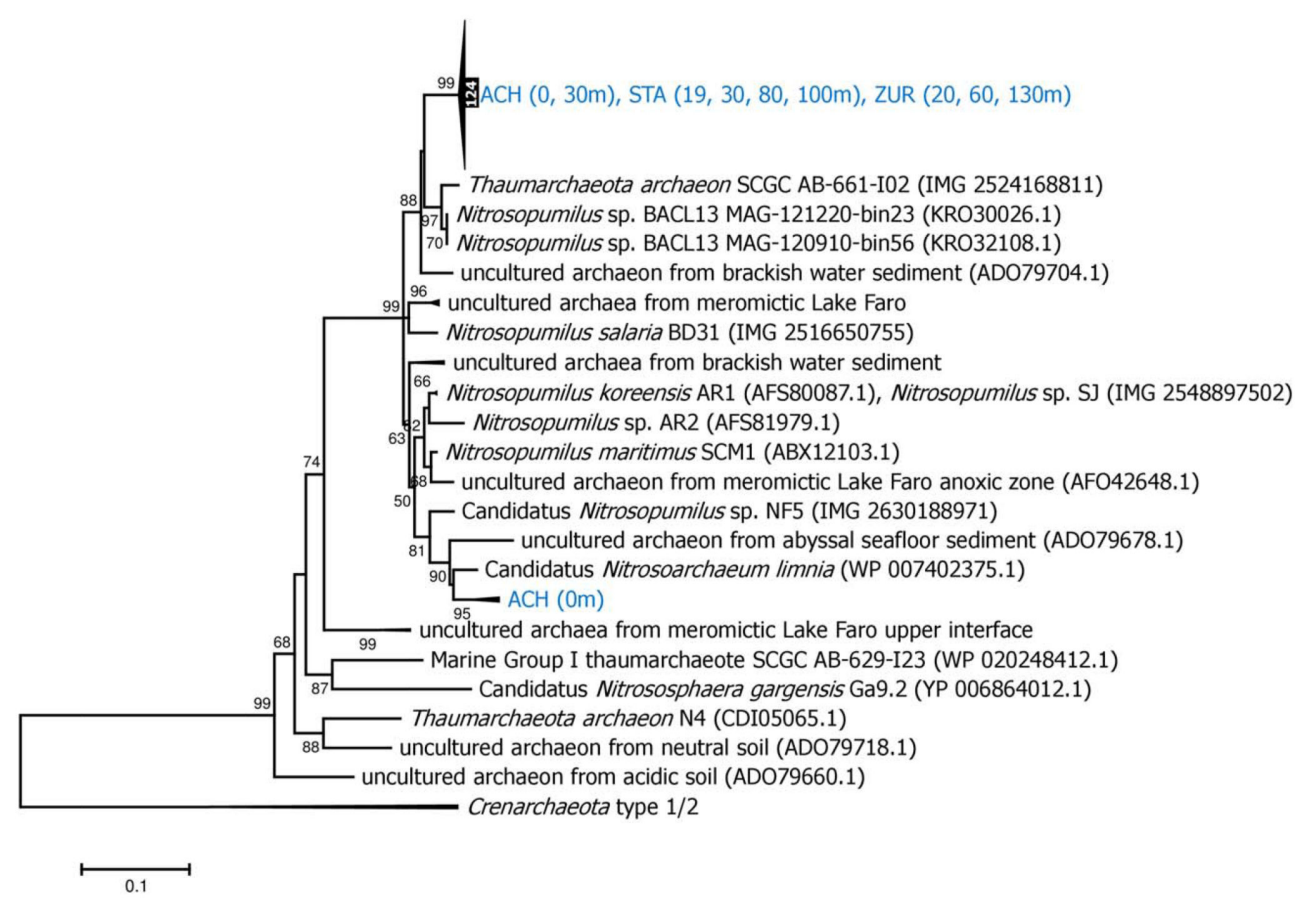
Neighbor-Joining trees obtained from deduced amino acid sequences of *hcd* genes in the HP/HB cycle derived from this study (shown in blue letters) and representative thaumarchaeal sequences from NCBI and IMG databases. Bootstrap values are shown as percentages of 1000 replicates and values over 50% are indicated on nodes. Scale bar indicates 10% changes in the amino acid sequence. [Color figure can be viewed at wileyonlinelibrary.com]

The *hcd* amino acid sequences, analysed from different depths in the large lakes ACH, STA and ZUR, revealed a major cluster of 124 highly similar sequences (Fig. 2). The closest relatives of this clade were found in a metagenome reconstructed from brackish water samples of the Baltic Sea (*Nitrosopumilus* sp. BACL13; Hugerth *et al*., 2015) and from a single cell genome analysed from marine Antarctic winter water at 80m depth (*Thaumarchaeota* archaeon SCGC AB-661-I02; Luo *et al*., 2014). The 16S rRNA of *Nitrosopumilus* sp. BACL13 showed a sequence similarity of 99% to marine lineages of *Nitrosopumilus maritimus*. The genomic repertoire of *Nitrosopumilus* sp. BACL13 also contained ammonia monooxygenase and urease genes, supporting an autotrophic lifestyle based on nitrification. The *Thaumarchaeota* archaeon SCGC AB-661-I02 genome belongs to members of an epipelagic clade of *Thaumarchaeota*, which harbour photolyase and catalase genes that are probably related to adaptive strategies to protect from light-induced damage (Luo *et al*., 2014). However, DNA photolyase was not specifically found in *Thaumarchaeota* archaeon SCGC AB-661-I02, which is inconclusive because of the low recovery (35%) of the genome sequence. Mechanisms to reduce light-induced damage could also have important implication for the presence of *Thaumarchaeota* in our study lakes, because several studies have shown that photoinhibition might be an important factor determining the distribution and relative contribution of ammonia oxidizing *Thaumarchaeota* in aquatic systems (Merbt *et al*., 2012). The second cluster of *hcd* sequences, which was exclusively detected in the surface water of ACH (0 m), was phylogenetically affiliated with uncultured thaumarchaeal clone sequences recovered from brackish water sediments and several ammonia oxidizing *Nitrosopumilaceae* isolates (Fig. 2).

**Fig. 3:**
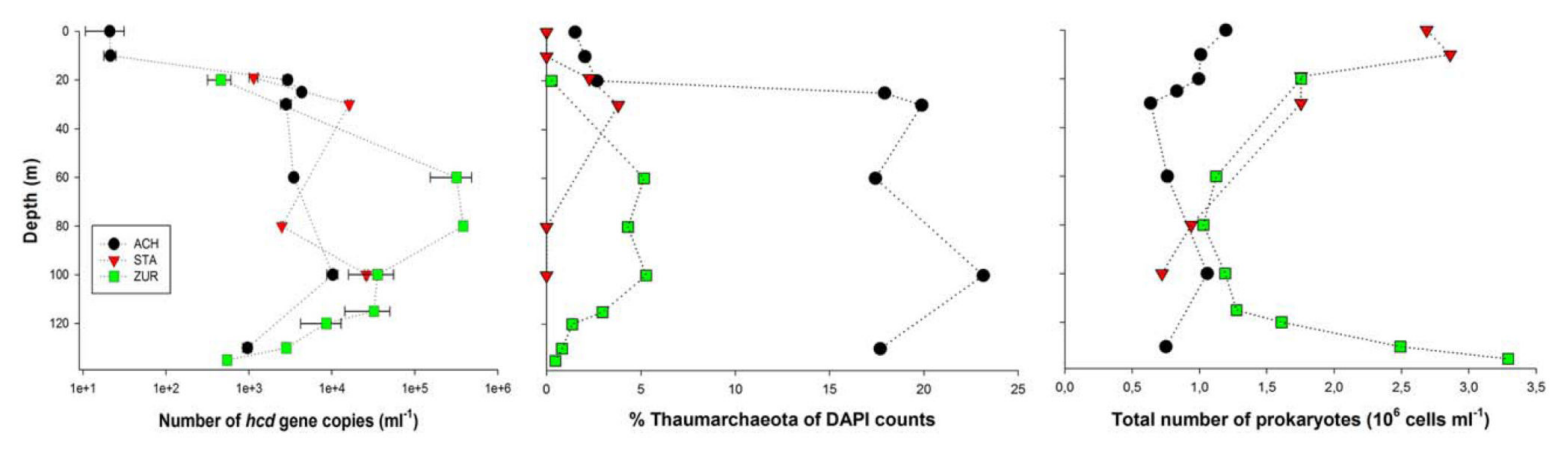
Depth profiles of *hcd* gene abundances (left plot), proportion of *Thaumarchaeota* (% of DAPI stained cells, central plot) and total number of prokaryotes (right plot) in lakes ACH, STA and ZUR. Gene abundances are based on three replicated qPCR analyses with standard deviations indicated by error bars. [Color figure can be viewed at wileyonlinelibrary.com]

Quantification of *hcd* genes by qPCR and the enumeration of *Thaumarchaeota* by CARD-FISH showed a similar distribution pattern between the lakes: the presence of *Thaumarchaeota* in large lakes and their absence in the smaller lakes EGE, HEC and PIB (Fig. 3), with the restriction that analysis in PIB did not include CARD-FISH counts. Vertical profiles of relative CARD-FISH numbers (i.e. percentages of total prokaryotic cell numbers) and *hcd* gene abundance showed the absence (STA) or a very low proportion (ACH) of *Thaumarchaeota* in the epilimnion of the large lakes, with a strong increase of their abundance in the hypolimnion (Fig. 3). The epilimnion of ZUR was not investigated, but CARD-FISH counts from another study during summer stratification showed that relative abundances of *Thaumarchaeota* in the surface water (3 and 10 m) of ZUR were below 1% (Callieri *et al*., 2014). In ZUR, *hcd* genes and relative abundances of *Thaumarchaeota* steadily decreased in the deeper hypolimnion, following the trend of oxygen depletion (Fig. 1, Supporting Information Table S2). In lake STA, CARD-FISH counts were below the quantification limit in 80 m and 100 m. These measurements were not supported by *hcd* gene copy numbers, which showed no decrease with depth (Fig. 3). Relative abundances of *Thaumarchaeota* were highest in the hypolimnion of ACH, with the maximum (23.1%) recorded in 100 m depth. However, the number of *hcd* genes was two magnitudes higher in ZUR with a maximum value of 0.384 x 10^6^ genes ml^−1^ in 80m depth (Fig. 3). One explanation for this discrepancy is the significant difference of the total number of prokaryotes and *Thaumarchaeota* detected in both lakes (Fig. 3), which are not reflected by the relative numbers. Different detection limits of the two approaches provide another explanation for the disagreement between qPCR results and CARD-FISH counts. Gene copy numbers detected by the qPCR method was measured as low as 20 genes ml^−1^ in lake ACH, while microscopic-based methodologies do not allow cell counts at this low magnitude. On the other hand, qPCR based quantification of individual genes is vulnerable to different methodological problems and biases whereas CARD-FISH counts are very reliable since they include the direct detection and quantification of single cells (Baptista *et al*., 2014). Accordingly, both quantification approaches used in our study could potentially lead to the over- or underrepresentation of *Thaumarchaeota* and their related *hcd* genes.

### Diversity and distribution of the rTCA cycle

**Fig. 4:**
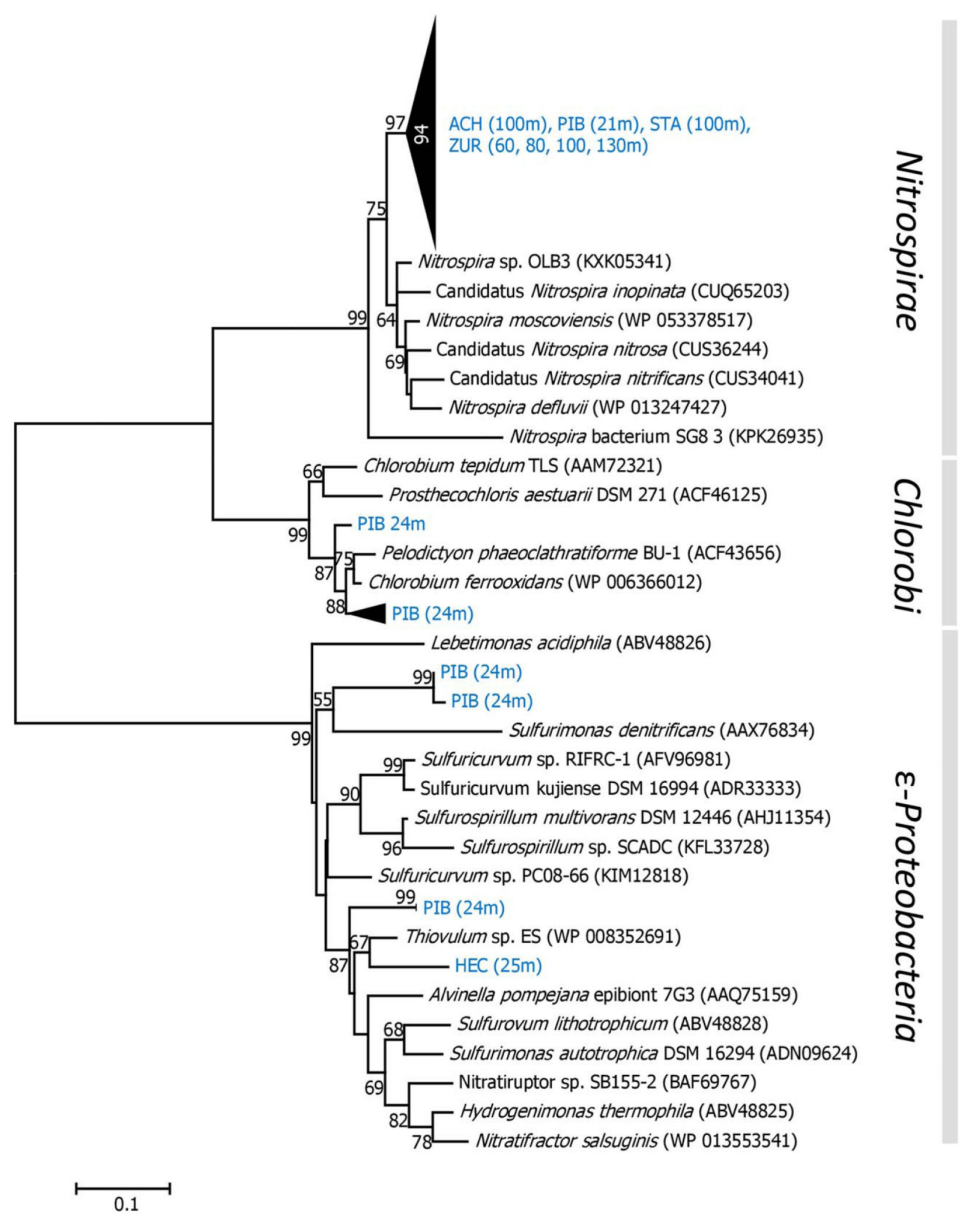
Neighbor-Joining trees obtained from deduced amino acid sequences of *aclA* genes in the rTCA pathway derived from this study (shown in blue letters) and representative sequences from NCBI and IMG databases. Bootstrap values are shown as percentages of 1000 replicates and values over 50% are indicated on nodes. Scale bar indicates 10% changes in the amino acid sequence. [Color figure can be viewed at wileyonlinelibrary.com]

Genes coding for the key enzyme ATP citrate lyase (*aclA*) in the rTCA cycle were successfully amplified from deep water of HEC (25 and 50 m) and PIB (21 and 24 m; Fig. 1), characterized by anoxic conditions. At all other sampling stations, the *aclA* genes were not detected based on a nested PCR approach (Supporting Information Table S1, see also chapter ‘optimization of the functional gene approach’). Phylogenetic analyses, performed with a limited amount of sequences obtained from two sampling stations (PIB 24 m, HEC 25 m), revealed that *aclA* genes were affiliated with *Epsilonproteobacteria* in both lakes (Fig. 4). The closest relatives were chemoautotrophic bacteria, including *Thiovulum* sp. in HEC and bacteria of the family *Helicobacteraceae* (*Sulfuricurvum* spp. and *Sulfurimonas denitrificans*). All of them are capable to extract energy from the oxidation of reduced sulfur compounds. Phylotypes related to green phototrophic *Chlorobi* were exclusively found in PIB.

A second primer pair was designed to specifically amplify genes coding for ATP citrate lyase in *Nitrospira*. *AclA* genes were detected in all deep lakes (ACH, STA, ZUR) and in the hypolimnion of PIB, while in water samples obtained from EGE and HEC marker genes for the rTCA cycle related to *Nitrospira* were not identified. Sequences of *aclA* genes retrieved from selected hypolimnion water samples of ACH, PIB, STA and ZUR formed a distinct cluster which was affiliated with different nitrite-oxidizing (*Nitrospira* sp. OLB3, *N. defluvii, N. moscoviensis*) and complete ammonia oxidizing (comammox) *Nitrospira* (Candidatus *N. inopinata*, Candidatus *N. nitrosa*, Candidatus *N. nitrificans*). Phylogenetic analyses also suggest that the ATP citrate lyase gene is not a suitable phylogenetic marker to differentiate between both metabolisms (Fig. 4). However, the differentiation between comammox and strict NOB is also not possible with other potential marker genes, such as *nxrB* and 16S rRNA genes (Pinto *et al*., 2016; Pjevac *et al*., 2016). Therefore, the functional role of *Nitrospira* populations in the nitrogen cycle at our study sites remains unresolved.

Two key factors are likely to create unique niches that favour the rTCA or the CBB cycle of bacteria at our study lakes: energy requirements of the pathways and the oxygen tolerance of key enzymes. In silico, biomass production based on CO_2_ assimilation is energetically less expensive with the rTCA cycle than the CBB pathway (Mangiapia and Scott, 2016). However, two key enzymes in the rTCA cycle, 2-oxoglutarate:ferredoxin oxidoreductase and pyruvate:ferredoxin oxidoreductase are generally known to be sensitive to oxygen. As a result, rTCA-based CO_2_ fixation mostly occurs in anaerobes, although special modifications of both enzymes to oxic conditions are known (Yamamoto *et al*., 2006). This includes microaerophilic bacteria, such as *Hydrogenobacter thermophilus* (Shiba *et al*., 1985), but recent investigations also suggest that similar enzymatic adaptations allow the rTCA cycle to function in members of the *Nitrospirae* phylum and the marine nitrite oxidizing bacteria *Nitrospina* (Lücker *et al*., 2010; 2013; Daims *et al*., 2015). In addition, some still unknown mechanisms may strengthen the O_2_ robustness of the rTCA pathway in aerobes; however, if this protection comes at the expense of a lower specific activity, it may significantly increase the energy demands of the cycle (Berg, 2011).

### Functional traits associated with different CO_2_ fixation pathways

**Fig. 5:**
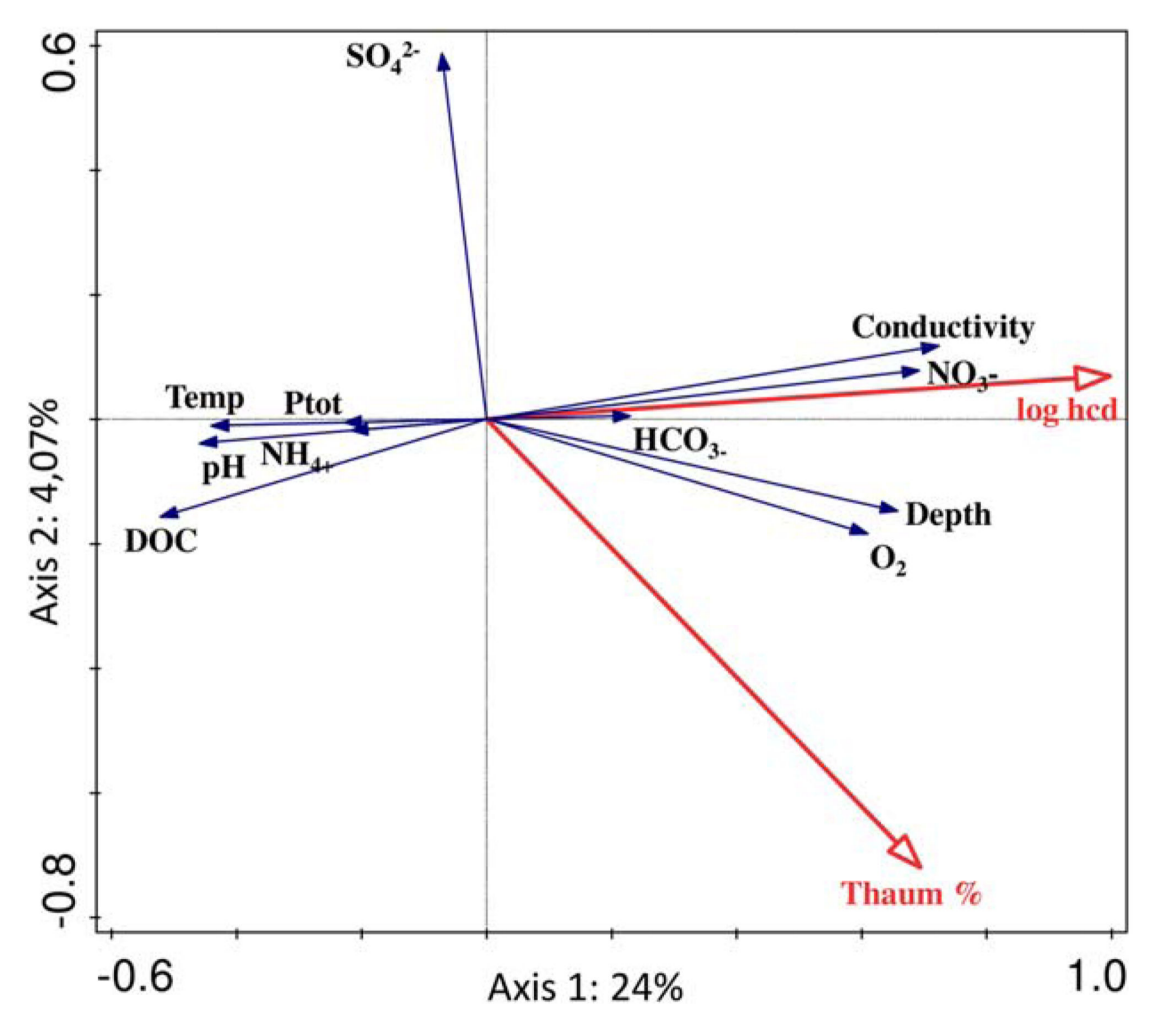
Redundancy analysis biplots of thaumarchaeotal proportions (Thaum %), log *hcd* gene copy numbers (log *hcd*) and selected environmental parameters from the studied lakes. [Color figure can be viewed at wileyonlinelibrary.com]

**Fig. 6:**
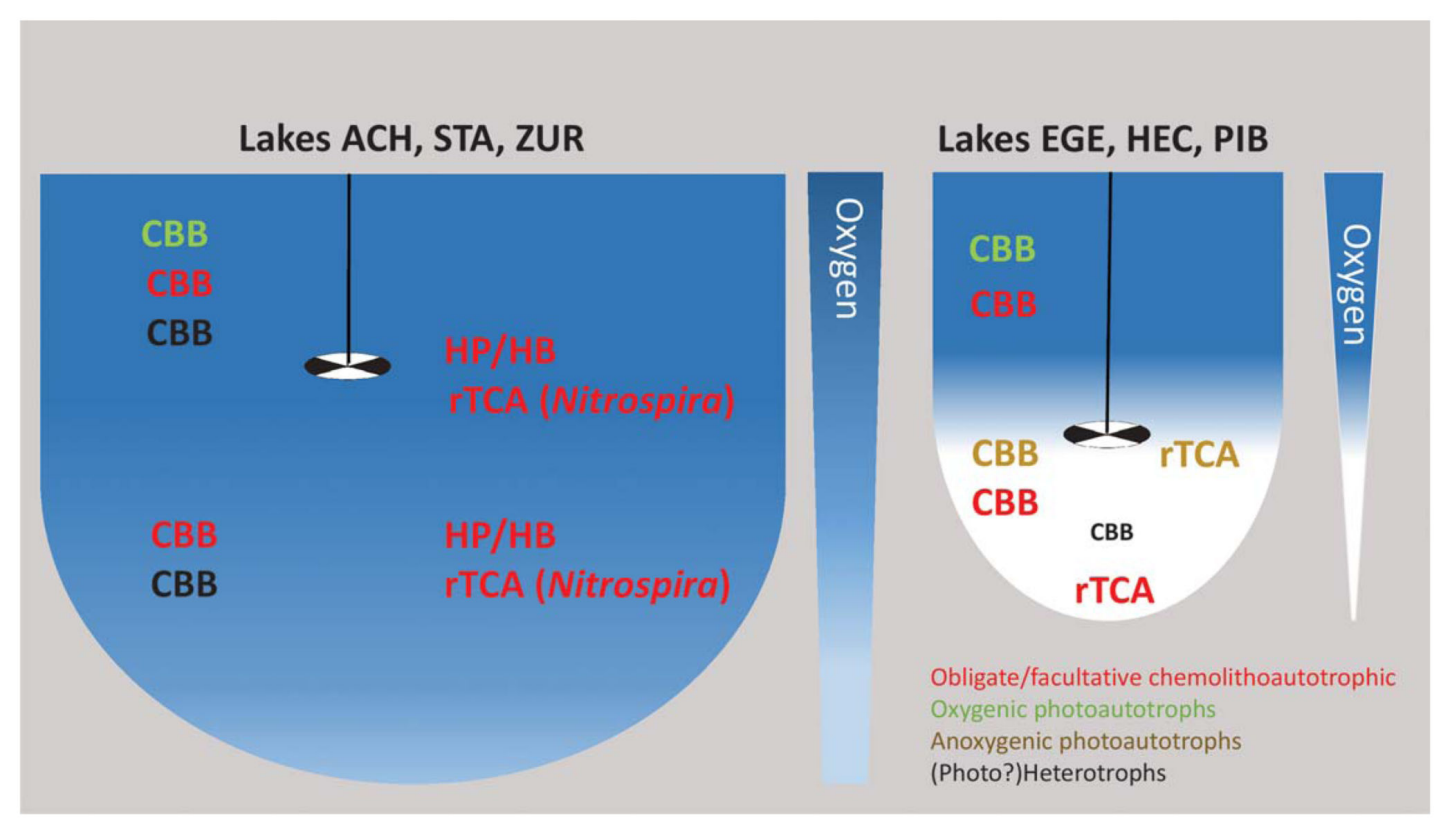
Generalized picture depicting different CO_2_ fixation strategies in the studied lakes.

Nitrification is a major source of energy for all three chemoautotrophic carbon fixation pathways observed at all lakes, with exception of EGE (Supporting Information Fig. S1A; Figs 2 and 4). Whereas bacteria, affiliated with representatives of the *Nitrosomonas* cluster 6a, assimilate CO_2_ with form IA RubisCO in the CCB cycle, functional genes in the HP/HB and rTCA cycles are dominated by phylotypes affiliated with nitrifying *Thaumarchaeota* and *Nitrospirae*. Notably, the recent discovery of comammox *Nitrospira* (Daims *et al*., 2015; van Kessel *et al*., 2015) has major implications for understanding the ecological niche of this bacterial group in various habitats, including lakes. However, these findings also limit our interpretation of the distribution of *Nitrospira* at our study sites, because based on the *aclA* phylogenies it is not determinable whether nitrite oxidation or complete ammonia oxidation is the primary lifestyle of *Nitrospira* using the rTCA cycle for CO_2_ assimilation (Fig. 4). For future research, the development of proper methods are needed to more efficiently detect and quantify different functional traits of *Nitrospira* in the environment. Another major question yet to be answered is why *Thaumarchaeota* using the HP/HB cycle were widely distributed in the hypolimnion of the large lakes ACH, STA, and ZUR, but were absent in EGE, HEC, and PIB. Although we cannot exclude the possibility that *Thaumarchaeota* occupy a narrow (temporal or/and spatial) niche that was missed by the sampling scheme, it is more likely that the actual environmental conditions determine their existence. Redundancy analysis (RDA) showing correlations between *hcd* gene abundances and selected environmental variables, indicates the highest relationship between nitrate, the final product of nitrification, conductivity and depth (Fig. 5). Dissolved organic carbon, pH and temperature, which are generally higher in the epilimnion, were negatively correlated. Ammonia as electron donor for *Thaumarchaeota* and total phosphorus correlated also negative, which supports the adaptation of those organisms to nutrient-limited conditions (Könneke *et al*., 2014). However, the direct effect of all these parameters on the growth of ammonia oxidizing *Archaea* in freshwater environments was also controversially discussed (Auguet *et al*., 2012; French *et al*., 2012; Berdjeb *et al*., 2013; Hugoni *et al*., 2013; La Cono *et al*. 2013, Vissers *et al*., 2013; Coci *et al*., 2015; Hugoni *et al*., 2015). The lack or low number of *Thaumarchaeota* and *hcd* genes in the surface water of the lakes may also be caused by additional factors related to the upper water column such as a competition with phototrophic microbes for small amounts of ${\text{NH}}_{4}^{+},$ heavy grazing pressure on slow-growing nitrifiers and/or inhibition by light (Small *et al*., 2013; Vissers *et al*., 2013). When these inhibiting components related to the euphotic zone overlap with the lack of oxygen in the hypolimnion, as is the case in lakes EGE, HEC, and PIB, environmental conditions are not suitable for *Thaumarchaeota* and could explain their absence in these lakes (Fig. 6). The community of nitrifiers using the CBB cycle were probably less affected, because ammonia oxidizers related to *Nitrosomonas* were detected in the metalimnion of lakes PIB (12 and 15 m) and HEC (14 m) (Supporting Information Fig. S1A). In these depths oxygen was still available and light dependent factors (light inhibition, competition by autotrophs) are reduced. EGE was the only lake were functional genes of all three CO_2_ fixation pathways were not related to nitrifying taxa. The reasons for their scarcity might be oxygen limitation at most sampling depths, but particularly the very high number of photoautotrophic organisms detected over the entire water column might outcompete chemolithoautotrophic ammonia oxidizers for ${\text{NH}}_{4}^{+}$ (Supporting Information Fig. S7). Certainly, further studies are required to decipher the niche differentiation of these ammonia-oxidizing guilds and to evaluate if their distribution is also linked to their different strategies for CO_2_ fixation. Although methodically demanding, also direct measurements of chemolithoautrophic CO_2_ fixation rates, ideally in combination with molecular approaches for the identification of the most important taxa, would be very helpful to assess the contribution of nitrifying representatives to the total CO_2_ uptake in lakes. The quantitative data observed in this study showed that *Thaumarchaeota* represent a considerable fraction of the prokaryotes detected over the entire water columns of the oxygenated hypolimnion in deep lakes (Fig. 3). Considering that photoautotrophic growth is being restricted to the euphotic zone, which accounts only for a small fraction of the entire water column in lakes ACH, STA and ZUR, our investigations confirm the results from previous studies (Callieri, 2016) that *Thaumarchaeota* contribute significantly to the carbon cycle in deep freshwater lakes.

Beside nitrification, obligate chemolithoautotrophs using reduced sulfur compounds as electron donor were observed in the anoxic water column of HEC and PIB, using either the rTCA cycle or forms IA/II RubisCO in the CBB cycle for the assimilation of CO_2_. The metabolic lifestyle of the closest relatives using Form IC and II RubisCO for CO_2_ fixation in the CBB cycle is diverse, but especially RubisCO *cbbL* IC phylotypes are often associated with facultative chemolithoautotrophs (Badger and Bek, 2008). The phylogenetic position of several *cbbL* IC sequences retrieved from ACH 129 m suggest that methylotrophic autotrophy is an important metabolism close to the bottom of the lake. In this instance, CO_2_ produced by the oxidation of an organic C1 compound is further assimilated via the CBB pathway, and not by the serine or ribulose monophosphate cycle (Baxter *et al*., 2002; Dedysh *et al*., 2005). This alternative type of nutrition based on RubisCO and the CBB cycle was also observed in a former study performed in polluted groundwater, where methylotrophic autotrophs play a key role for aerobic methyl tert-butyl ether degradation in contaminated aquifers (Alfreider *et al*., 2012).

Several *cbbL* and *cbbM* sequences were affiliated with bacteria that are known for their photoheterotrophic or heterotrophic lifestyle (Supporting Information Fig. S1B and C). In these organisms, the CBB cycle is probably required as an electron sink during growth on substrates that are more reduced than the average cell carbon (Ferguson *et al*., 1987). It has been suggested that this electron-accepting process is not restricted to anoxygenic phototrophs (also known as purple non-sulfur bacteria), the use of CO_2_ fixation to maintain redox balance would also be useful for nonphotosynthetic heterotrophic bacteria growing under hypoxic conditions (McKinlay and Harwood, 2010).

## Experimental procedures

### Sampling sites and collection of lake water samples

Six lakes were selected based on their physical and chemical characteristics, especially the differences in their stratification patterns (Table 1 and Supporting Information Fig. S6). Samples were taken during summer or late summer stratification. The geographic location and the main limnological and morphometric parameters of the study sites are shown in Table 1.

Prior to sampling, water column *in situ* profiles of dissolved oxygen, temperature and the redox potential were measured with a multi-parameter probe (YSI model 6600; Yellow Springs Instruments, OH) to identify stratification gradients. Based on these data, eight to ten samples were taken along the water column at the deepest part of the lakes, with an increased sampling resolution along pronounced oxygen gradients (Supporting Information Table S2, Fig. 1). At EGE and ZUR, the epilimnion was not included in the sampling scheme. Samples were taken with a modified Schindler-Patalas sampler in HEC, PIB and ZUR. A modified Ruttner sampler, equipped with a messenger-controlled closing mechanism, was used for sampling ACH and STA. At EGE, where pronounced stratification causes strong gradients in the water chemistry, a tube water sampling device was used to take discrete samples at distances in the order of centimetres along the pelagic redoxcline. From each sampling depth, between one to 2 l of lake water were filled into sterile glass bottles for biological analysis and 1 l of water was collected into separate flasks for chemical analysis.

Chemical analysis of lake water obtained from selected sampling depths were carried out at two laboratories (ZUR water samples by the Zurich Water Supply Company, all other lakes at the Institute of Ecology, University of Innsbruck). Major anion and cation compositions of the lake water samples were analysed by ion chromatography (Dionex DX-120, Dionex Inc., Sunnyvale, CA). Total organic carbon (TOC) and dissolved organic carbon (DOC) were determined using a TOC/TN-Analyzer (TOC-5000, Shimadzu, Kyoto, Japan). In addition to oxygen probe measurements, parallel determination of oxygen concentration at sampling depths were carried out using the Winkler titration method.

### DNA-extraction, PCR and cloning

For DNA analyses, 300–1100 ml of lake water was collected on polyethersulfone filters (pore size 0.22 μm, Millipore, Bedford, MA) in the field, immediately frozen on dry ice and stored in the laboratory at −20°C until use. DNA extraction was performed with the PowerWater® DNA isolation kit (Mo Bio Laboratories, Inc., Carlsbad, CA) according to the manufacturer’s protocol. Supplementary methods section and Supporting Information Table S1 summarizes the specification of several sets of oligonucleotide primers, which were used for PCR amplification of genes coding for key enzymes of different CO_2_ fixation pathways. PCR was performed with the HotStarTaq® *Plus* Master Mix Kit (Qiagen Inc., Valencia, CA) following the manufacturer’s instructions. The annealing temperatures used for individual primer sets are listed in Supporting Information Table S1. A nested PCR-approach with two broad range primers was used for the amplification of the *aclA* genes (Supporting Information Table S1). One μl of PCR-products produced with primers aclA_f680/aclA_r1491 after 20 cycles was used as template for a second PCR-reaction with the primer set aclA_f807/aclA_r1371 (35 cycles). All PCR products selected for sequencing analysis were separated on 1.5% agarose gels. Bands with proper size were selected for subsequent cloning, cut out of the gel and purified using either the Wizard® SV Gel and PCR Clean-Up system (Promega Corporation, Madison, WI) or the MinElute® Gel Extraction Kit (Qiagen Inc., Valencia, CA). PCR products were ligated into pGEM-T-Easy Vector plasmid (Promega, Madison, WI) and transformed into JM109 competent cells following the manufacturer’s instructions. Clones were screened for the presence of proper inserts by PCR using vector-specific primers M13-F/R and GoTaq® G2 Hot Start Master Mix (Promega, Madison, WI) following the protocol provided by the manufacturer. Selected reactions were Sanger sequenced by a sequencing service enterprise (Eurofins MWG Operon, Ebersberg, Germany).

### Quantitative PCR of *hcd* genes

DNA concentrations in lake water samples were measured using a Quantus fluorometer (Promega Corporation, Madison, WI) and QuantiFluor® dsDNA chemistry (Promega Corporation, Madison, WI) according to the protocols recommended by the manufacturer. Thaumarchaeal *hcd* genes were analysed by quantitative PCR using primers specifically designed for this study (Supporting Information Table S1). The optimal annealing temperature was determined empirically with DNA from water samples of lakes ACH, STA and ZUR (Supporting Information Fig. S4). Samples from PIB, which produced no PCR-Products with *hcd* broad range primers (Fig. 1), were used as negative control. Furthermore, the specificity and coverage of the primer pair was evaluated by sequence analysis after cloning *hcd* genes amplified from ACH, PIB, STA as described above applying an annealing temperature of 56°C (Supporting Information Fig. S5). All qPCR analyses were done on an ABI 7300 system (Applied Biosystems, Foster City, CA). The reactions were performed in triplicate in 96 well white qPCR plates. Twenty μl reaction mixtures contained 10 μl Power SYBR® Green PCR Master Mix (Applied Biosystems, Carlsbad, CA), 0.2 mmol l^−1^ of forward and reverse primers, 2μl template DNA (2–10ng) and molecular biology-grade water. The reactions had an initial denaturing step of 10 min at 95°C, followed by 35 cycles including 15 s denaturing at 95°C, 15 s of primer annealing at 56°C and 45 s of primer extension at 60°C. Primer specificity was assessed by melt curve analysis and confirmed by running amplicons on agarose gel electrophoresis. Standard curves for quantification were based on 10-fold dilutions ranging from 10^1^ to 10^8^ copies of DNA of a *hcd* amplicon of an environmental clone selected from clone libraries produced for the diversity studies (procedure see above). A GeneJET Plasmid Miniprep Kit (Thermo Scientific, Waltham, MA) was used to extract the plasmids, which were linearized with BcuI restriction enzyme (Thermo Scientific, Waltham, MA), purified with a Qiaquick PCR Purification Kit (Qiagen Inc., Valencia, CA) and measured fluorometrically (Quantus fluorometer, Promega, Madison, WI).

### CARD-FISH of *Thaumarchaeota*

CARD-FISH of water samples from ZUR, EGE, ACH, HEC and STA was done following the protocol of Wendeberg (2010). In short, samples were fixed with 0.2 μm filtered form-aldehyde (2% final concentration) and aliquots of 5–15 ml were filtered onto 0.2 μm white polycarbonate filters (Poretics, 47 mm filter diameter) and embedded in 0.1% ultrapure agarose (wt./vol., SeaKem® LE Agarose, Lonza, Basel, Switzerland). Filters were digested with lysozyme and proteinase K and hybridized with horseradish peroxidase (HRP)-labelled oligonucleotide probes. We used oligonucleotide probe MGI-535 specifically targeting *Thaumarchaeota* (Coci *et al*., 2015). Stringent hybridization conditions were achieved with 45% formamide in the hybridization buffer. Alexa488 tyramides were used for signal amplification. The filters were counterstained with 4′,6-Diamidin-2-phenylindol (DAPI) and embedded in a 5:1:1 mix of Citifluor (Citifluor Ltd., London), Vectashield (Vector Laboratories, Inc., Burlingame, CA) and PBS. At least 400 DAPI positive cells (minimum 15 CARD-FISH positive cells) were counted with a Zeiss Axioplan epifluorescence microscope.

### Statistics and phylogenetic analysis

The software package CANOCO 5 (Šmilauer and Lepš, 2014) was used for redundancy analysis (RDA) of environmental parameters with *hcd* gene abundances and CARD-FISH counts of *Thaumarchaeota*. The gene abundances were log-transformed to create normal distributions. Significance of RDA was determined using Monte-Carlo permutations as recommended in the programme documentation.

Closest relatives to nucleotide sequences and deduced amino acid sequences were obtained using NCBI’s sequence similarity search tools BLASTN and BLASTP (http://www.ncbi.nlm.nih.gov/BLAST/) and the BLAST algorithm within the IMG/M analysis system for metagenomes (https://img.jgi.doe.gov/). Deduced amino acids were aligned using Clustal W or MUSCLE algorithm as implemented in MEGA 6.0 software (Tamura *et al*., 2013), followed by visual inspection of the alignment. Neighbor-Joining trees applying gamma distribution as the distance method were also computed with the MEGA 6 software package. Bootstrap analysis (1000 replicates) was used to obtain confidence estimates for tree topology. The phylogenetic tree was condensed by compressing subtrees with highly similar sequences.

Sequences data have been submitted to GenBank databases under accession numbers KY418180 – KY418605 (*cbbL*-Form IA), KY447334 – KY447878 (*cbbL*-Form IC), KY447879 – KY448163 (*cbbM*), KY448164 – KY448240 (*hcd*), KY458233 – KY458281 (*hcd*), KY458282 – KY458375 (*acl-Nitrospira*), KY458376 – KY458390 (*acl*).

## Supporting information

Additional Supporting Information may be found in the online version of this article at the publisher’s website:

Supplementary Information

## Figures and Tables

**Table 1:** Main characteristics of the studied lakes.

Parameter	Egelsee (EGE)	Piburger See (PIB)	Hechtsee (HEC)	Achensee (ACH)	Starnberger See (STA)	Zürichsee (ZUR)
Location	47.40057° N	47.19492° N	47.60908° N	47.45694° N	47.91667° N	47.25778° N
	8.359646° E	10.88875° E	12.16193° E	11.70861° E	11.31667° E	8.64889° E
Sampling date	22 Sep 2010	01 Jul 2013	07 Aug 2013	17 Oct 2013	30 Aug 2013	21 Sep 2010
Altitude (m a.s.l.)	667	913	542	929	590	406
Area (km^2^)	0.02	0.134	0.28	6.8	56.36	66.82
Max depth (m)	10.5	24.6	57	133	128	136
Mixing type	Dimictic	Dimictic	Meromictic	Dimictic	Monomictic	Monomictic
Trophic State	Mesotrophic	Oligo-mesotrophic	Mesotrophic	Ultra-oligotrophic	Oligo-mesotrophic	Mesotrophic

Abbreviation of lake names used in the main text are shown in parenthesis.

## References

[R1] Alfreider A, Schirmer M, Vogt C (2012). Diversity and expression of different forms of RubisCO genes in polluted groundwater under different redox conditions. FEMS Microbiol Ecol.

[R2] Alfreider A, Vogt C, Kaiser M, Psenner R (2009). Distribution and diversity of autotrophic bacteria in groundwater systems based on the analysis of RubisCO genotypes. System Appl Microbiol.

[R3] Auguet JC, Borrego CM, Bañeras L, Casamayor EO (2008). Fingerprinting the genetic diversity of the biotin carboxylase gene (*accC*) in aquatic ecosystems as a potential marker for studies of carbon dioxide assimilation in the dark. Environ Microbiol.

[R4] Auguet J-C, Triado-Margarit X, Nomokonova N, Camamero L, Casamayor EO (2012). Vertical segregation and phylogenetic characterization of ammonia-oxidizing archaea in a deep oligotrophic lake. ISME J.

[R5] Badger MR, Bek EJ (2008). Multiple Rubisco forms in proteobacteria: their functional significance in relation to CO_2_ acquisition by the CBB cycle. J Exp Bot.

[R6] Baptista JDC, Lunn M, Davenport RJ, Swan DL, Read LF, Brown MR (2014). Agreement between amoA gene-specific quantitative PCR and fluorescence *in situ* hybridization in the measurement of ammonia-oxidizing bacteria in activated sludge. Appl Environ Microbiol.

[R7] Baxter NJ, Hirt RP, Bodrossy L, Kovacs KL, Embley TM, Prosser JI, Murrell JC (2002). The ribulose-1,5-bisphosphate carboxylase/oxygenase gene cluster of *Methylococcus capsulatus* (Bath). Arch Microbiol.

[R8] Berdjeb L, Pollet T, Chardon C, Jacquet S (2013). Spatiotemporal changes in the structure of archaeal communities in two deep freshwater lakes. FEMS Microbiol Ecol.

[R9] Berg IA (2011). Ecological aspects of the distribution of different autotrophic CO_2_ fixation pathways. Appl Environ Microbiol.

[R10] Bergauer K, Sintes E, Bleijswijk JV, Witte H, Reinthaler T, Herndl GJ (2013). Abundance and distribution of archaeal acetyl-CoA/propionyl-CaA carboxylase genes indicative for putatively chemoautotrophic Archaea in the tropical Atlantic’s interior. FEMS Microb Ecol.

[R11] Bollmann A, Sedlacek CJ, Norton J, Laanbroek HJ, Suwa Y, Stein LY (2013). Complete genome sequence of *Nitrosomonas* sp. Is79, an ammonia oxidizing bacterium adapted to low ammonium concentrations. Stand Genomic Sci.

[R12] Bouskill NJ, Eveillard D, Chien D, Jayakumar A, Ward BB (2012). Environmental factors determining ammonia-oxidizing organism distribution and diversity in marine environments. Environ Microbiol.

[R13] Callieri C (2016). Micro-players for macro-roles: aquatic microbes in deep lakes. J Limnol.

[R14] Callieri C, Coci M, Eckert EM, Salcher MM, Bertoni R (2014). Archaea and bacteria in deep lake hypolimnion: in situ dark inorganic carbon uptake. J Limnol.

[R15] Casamayor EO (2010). Vertical distribution of planktonic auto-trophic thiobacilli and dark CO_2_ fixation rates in lakes with oxygen-sulfide interfaces. Aquat Microb Ecol.

[R16] Coci M, Odermatt N, Salcher M, Pernthaler J, Corno G (2015). Ecology and distribution of Thaumarchaea in the deep hypolimnion of Lake Maggiore. Archaea.

[R17] Daims H, Lebedeva EV, Pjevac P, Han P, Herbold C, Albertsen M (2015). Complete nitrification by *Nitrospira* bacteria. Nature.

[R18] Daims H, Lücker S, Wagner M (2016). A new perspective on microbes formerly known as nitrite-oxidizing bacteria. Trends Microbiol.

[R19] Dedysh SN, Smirnova KV, Khmelenina VN, Suzina NE, Liesack W, Trotsenko YA (2005). Methylotrophic autotrophy in *Beijerinckia mobilis*. J Bacteriol.

[R20] Ferguson SJ, Jackson JB, McEwan AG (1987). Anaerobic respiration in the *Rhodospirillaceae*: characterization of pathways and evaluation of roles in redox balancing during photosynthesis. FEMS Microbiol Rev.

[R21] Field CB, Behrenfeld MJ, Randerson JT, Falkowski P (1998). Primary production in the biosphere: Integrating terrestrial and oceanic components. Science.

[R22] Fixen KR, Starkenburg SR, Hovde BT, Johnson SL, Deodato CR, Daligault HE (2016). Genome sequences of eight bacterial species found in coculture with the haptophyte *Chrysochromulina tobin*. Genome Announc.

[R23] French E, Kozlowski JA, Mukherjee M, Bullerjahn G, Bollmann A (2012). Ecophysiological characterization of ammonia-oxidizing archaea and bacteria from freshwater. Appl Environ Microbiol.

[R24] Giri BJ, Bano N, Hollibaugh J (2004). Distribution of RuBisCO genotypes along a redox gradient in Mono Lake California. Appl Environ Microbiol.

[R25] Herndl GJ, Reinthaler T (2013). Microbial control of the dark end of the biological pump. Nature Geosci.

[R26] Hu A, Yang Z, Yu C-P, Jiao N (2013). Dynamics of autotrophic marine planktonic *Thaumarchaeota* in the East China Sea. PLoS One.

[R27] Hugerth LW, Larsson J, Alneberg J, Lindh MV, Legrand C, Pinhassi J (2015). Metagenome-assembled genomes uncover a global brackish microbiome. Genome Biol.

[R28] Hügler M, Sievert SM (2011). Beyond the Calvin Cycle: autotrophic carbon fixation in the ocean. Ann Rev Mar Sci.

[R29] Hügler M, Wirsen CO, Fuchs G, Taylor CD, Sievert SM (2005). Evidence for autotrophic CO_2_ fixation via the reductive tricarboxylic acid cycle by members of the ∈ subdivision of proteobacteria. J Bacteriol.

[R30] Hugoni M, Domaizon I, Taib N, Biderre-Petit C, Agogué H, Galand PE, Debroas D, Mary I (2015). Temporal dynamics of active Archaea in oxygen-depleted zones of two deep lakes. Environ Microbiol Rep.

[R31] Hugoni M, Etien S, Bourges A, Lepère C, Domaizon I, Mallet C (2013). Dynamics of ammonia-oxidizing archaea and bacteria in contrasted freshwater ecosystems. Res Microbiol.

[R32] Kojima H, Fukui M (2010). *Sulfuricella denitrificans* gen. nov., sp. nov., a sulfur-oxidizing autotroph isolated from a freshwater lake. Int J Syst Evol Microbiol.

[R33] Kong W, Chuchiolo A, Priscu JC, Morgan-Kiss RM (2012a). Evidence of form II RubisCO (*cbbM*) in a perennially ice-covered Antarctic lake. FEMS Microbiol Ecol.

[R34] Kong W, Ream DC, Priscu JC, Morgan-Kiss RM (2012b). Diversity and expression of RubisCO genes in a perennially ice-covered Antarctic lake during the polar night transition. Appl Environ Microbiol.

[R35] Könneke M, Schubert DM, Brown PC, Hügler M, Standfest S, Schwander T (2014). Ammonia-oxidizing archaea use the most energy efficient aerobic pathway for CO_2_ fixation. Proc Natl Acad Sci USA.

[R36] Kovaleva OL, Tourova TP, Muyzer G, Kolganova TV, Sorokin DY (2011). Diversity of RuBisCO and ATP citrate lyase genes in soda lake sediments. FEMS Microbiol Ecol.

[R37] La Cono V, La Spada G, Arcadi E, Placenti F, Smedile F, Ruggeri G (2013). Partaking of Archaea to bio-geochemical cycling in oxygen-deficient zones of meromictic saline Lake Faro (Messina, Italy). Environ Microbiol.

[R38] Lücker S, Wagner M, Maixner F, Pelletier E, Koch H, Vacherie B (2010). A *Nitrospira metagenome* illuminates the physiology and evolution of globally important nitrite-oxidizing bacteria. Proc Natl Acad Sci USA.

[R39] Lücker S, Nowka B, Rattei T, Spieck E, Daims H (2013). The genome of *Nitrospina gracilis* illuminates the metabolism and evolution of the major marine nitrite oxidizer. Front Microbiol.

[R40] Luo H, Tolar BB, Swan BK, Zhang CL, Stepanauskas R, Moran MA, Hollibaugh JT (2014). Single-cell genomics shedding light on marine *Thaumarchaeota* diversification. ISME J.

[R41] Mangiapia M, Scott K (2016). From CO_2_ to cell: energetic expense of creating biomass using the Calvin-Benson-Bassham and reductive citric acid cycles based on genome data. FEMS Microbiol Lett 2016.

[R42] Martinez-Garcia M, Swan BK, Poulton NJ, Gomez ML, Masland D, Sieracki ME, Stepanauskas R (2012). High-throughput single-cell sequencing identifies photoheterotrophs and chemoautotrophs in freshwater bacterioplankton. ISME J.

[R43] McKinlay JB, Harwood CS (2010). Carbon dioxide fixation as a central redox cofactor recycling mechanism in bacteria. Proc Natl Acad Sci USA.

[R44] Merbt S, Stahl DA, Casamayor EO, Eugenia M, Nicol GW, Prosser JI (2012). Differential photoinhibition of bacterial and archaeal ammonia oxidation. FEMS Microbiol Lett.

[R45] Middelburg J (2011). Chemoautotrophy in the ocean. Geophys Res Lett.

[R46] Molari M, Manini E, Dell’Anno A (2013). Dark inorganic carbon fixation sustains the functioning of benthic deep-sea ecosystems. Global Biogeochem Cycles.

[R47] Noguerola I, Picazo A, Lliros M, Camacho A, Borrego CM (2015). Diversity of freshwater *Epsilonproteo-bacteria* and dark inorganic carbon fixation in the sulphidic redoxcline of a meromictic karstic lake. FEMS Microbiol Ecol.

[R48] Penton CR, Johnson T, Quensen J, Tiedje J (2013). Functional genes to assess nitrogen cycling and aromatic hydrocarbon degradation: primers and processing matter. Front Microbiol.

[R49] Pinto AJ, Marcus DN, Ijaz UZ, Bautista-de Lose Santos QM, Dick GJ, Raskin L (2016). Metagenomic evidence for the presence of comammox *Nitrospira*-like bacteria in a drinking water system. mSphere.

[R50] Pjevac P, Schauberger C, Poghosyan L, Herbold CW, van Kessel MAHJ, Daebeler A (2016). AmoA-targeted polymerase chain reaction primers for the specific detection and quantification of comammox *Nitrospira* in the environment. bioRxiv.

[R51] Raven JA (2009). Contributions of anoxygenic and oxygenic phototrophy and chemolithotrophy to carbon and oxygen fluxes in aquatic environments. Aquat Microb Ecol.

[R52] Seong HS, Soon DL (2009). *Actinoplanes subtropicus* sp. nov., isolated from rhizosphere soil. International Meeting of the Microbiological Society of Korea.

[R53] Shiba H, Kawasumi T, Igarashi Y, Kodama T, Minoda Y (1985). The CO_2_ assimilation via the reductive tricarboxylic-acid cycle in an obligately autotrophic, aerobic hydrogen-oxidizing bacterium, *Hydrogenobacter thermophilus*. Arch Microbiol.

[R54] Small GE, Bullerjahn GS, Sterner RW, Beall BFN, Brovold S, Finlay JC (2013). Rates and controls of nitrification in a large oligotrophic lake. Limnol Oceangr.

[R55] Šmilauer P, Lepš J (2014). Multivariate Analysis of Ecological Data Using CANOCO5.

[R56] Suwa Y, Norton JM, Bollmann A, Klotz MG, Stein LY, Laanbroek HJ (2011). Genome sequence of *Nitrosomonas* sp. strain AL212, an ammonia-oxidizing bacterium sensitive to high levels of ammonia. J Bacteriol.

[R57] Swan BK, Martinez-Garcia M, Preston CM, Sczyrba A, Woyke T, Lamy D (2011). Potential for chemolithoautotrophy among ubiquitous bacteria lineages in the dark ocean. Science.

[R58] Tabita FR, Satagopan S, Hanson TE, Kreel NE, Scott SS (2008). Distinct form I, II, III, and IV Rubisco proteins from the three kingdoms of life provide clues about Rubisco evolution and structure/function relationships. J Exp Bot.

[R59] Tamura K, Stecher G, Peterson D, Filipski A, Kumar S (2013). MEGA6: Molecular Evolutionary Genetics Analysis Version 6.0. Mol Biol Evol.

[R60] Tolar B, King GM, Hollibaugh JT (2013). An analysis of *Thaumarchaeota* populations from the Northern Gulf of Mexico. Front Microbiol.

[R61] Tourova TP, Kovaleva OL, Sorokin DY, Muyzer G (2010). Ribulose-1,5-bisphosphate carboxylase/oxygenase genes as a functional marker for chemolithoautotrophic halophilic sulfur-oxidizing bacteria in hypersaline habitats. Microbiol-UK.

[R62] van Kessel MA, Speth DR, Albertsen M, Nielsen PH, Op den Camp HJ, Kartal B (2015). Complete nitrification by a single microorganism. Nature.

[R63] Vissers EW, Anselmetti FS, Bodelier PL, Muyzer G, Schleper C, Tourna M, Laanbroek HJ (2013). Temporal and spatial coexistence of archaeal and bacterial amoA genes and gene transcripts in Lake Lucerne. Archaea.

[R64] Watanabe T, Kojima H, Fukui M (2012). Draft genome sequence of a psychrotolerant sulfur-oxidizing bacterium, *Sulfuricella denitrificans* skB26, and proteomic insights into cold adaptation. Appl Environ Microbiol.

[R65] Wendeberg A (2010). Fluorescence in situ hybridization for the identification of environmental microbes. Cold Spring Harb Protoc.

[R66] Wilhelm SW, Bullerjahn GS, Eldridge ML, Rinta-Kanto JM, Poorvin L, Bourbonniere RA (2006). Seasonal hypoxia and the genetic diversity of prokaryote populations in the central basin hypolimnion of Lake Erie: evidence for abundant cyanobacteria and photosynthesis. J Great Lakes Res.

[R67] Xu Q, Tabita FR (1996). Ribulose-1,5-bisphosphate carboxylase/oxygenase gene expression and diversity of Lake Erie planktonic microorganisms. Appl Environ Microbiol.

[R68] Yakimov MM, La Cono V, Denaro R (2009). A first insight into the occurrence and expression of functional *amoA* and *accA* genes of autotrophic and ammonia oxidizing bathypelagic *Crenarchaeota* of Tyrrhenian Sea. Deep Sea Res.

[R69] Yakimov MM, La Cono V, Smedile F, Deluca TH, Juárez S, Ciordia S (2011). Contribution of crenarchaeal autotrophic ammonia oxidizers to the dark primary production in Tyrrhenian deep waters (Central Mediterranean Sea). ISME J.

[R70] Yamamoto M, Arai H, Ishii M, Igarashi Y (2006). Role of two 2-oxoglutarate:ferredoxin oxidoreductases in *Hydrogenobacter thermophilus* under aerobic and anaerobic conditions. FEMS Microbiol Lett.

[R71] Yoshizawa Y, Toyoda K, Arai H, Ishii M, Igarashi Y (2004). CO_2_-responsive expression and gene organization of three ribulose-1,5-bisphosphate carboxylase/oxygenase enzymes and carboxysomes in *Hydrogenovibrio marinus* strain MH-110. J Bacteriol.

