# Supplementary material for: CO2 assimilation strategies in stratified lakes: Diversity and distribution patterns of chemolithoautotrophs
Source: Environ Microbiol. Author manuscript; Available in PMC 2017 Sep 28. (PMC5619642; doi:10.1111/1462-2920.13786)
Supplement: Supplementary Information [file NIHMS74255-supplement-Supplementary_Information.pdf]

Supplementary Information

CO_2 assimilation strategies in stratified lakes: diversity and distribution patterns of chemolithoautotrophs

A. Alfreider, A. Baumer, T. Bogensperger, T. Posch, M. M. Salcher and M. Summerer

Supplementary Methods

Optimization of the functional gene approach

Flow cytometry

Supporting Tables and Figures

Table S1

Specification of primers applied for the detection of different CO_2 fixation pathways

Table S2

Summary of physico-chemical parameters in the studied lakes

Fig. S1

Neighbor-Joining trees obtained from deduced amino acid sequences of forms IA (**Fig. S1A**), IC (**Fig. S1B**) and II (**Fig. S1C**) RubisCO genes in the CBB cycle

Fig. S2

Coverage of two different primer sets targeting Form II RubisCO sequences

Fig. S3

Coverage of two different primer sets targeting Form IC RubisCO sequences

Fig. S4

Evaluation of different annealing temperatures for thaumarchaeal *hcd* qPCR primers

Fig. S5

Evaluation of the specificity of *hcd* qPCR primers

Fig. S6

Vertical profiles of ammonium and nitrate in the studied lakes

Fig. S7

Vertical profile of autotrophic cell numbers in Lake Egelsee

Supplementary References (not included in main text)

Supplementary Methods

Optimization of the functional gene approach

In this study, a major effort was undertaken to (re)evaluate already published PCR-based marker systems and to design and test new primer pairs before they were applied on a routine basis at our study sites. Table S1 summarizes the specification of primers that were used. Not included in this table are other published primer pairs that were tested (in silico and/or in vitro), but were not appropriate for the investigation of lake water samples from our study sites.

Three sets of oligonucleotide primers were designed and successfully used for PCR and RT-PCR amplification of RubisCO form IA, form IC and form II gene fragments as applied in groundwater samples by Alfreider *et al.* (2003, 2009, 2012) and in exploratory studies in selected lakes (data not shown). Nonetheless, the specificity and coverage of these primers was evaluated based on new RubisCO sequences available in public sequence databases and recently published primer sets. For example**,** Kato *et al.* (2012) designed a PCR primer set targeting RubisCO form II gene where the coverage (in silico) was reported to be superior compared with the primers designed by Alfreider *et al.* (2003). However, in vivo validation of both primers systems revealed a different picture (Fig. S2) and individual primer sets covered only a part of the *cbbM* diversity observed in our study sites. In order to increase the coverage of *cbbM* genes, selected lake water samples were amplified with both primers sets for sequence analysis. For the same reason, two primers pairs were also used to study the diversity of the form IC RubisCO (Table S1. Fig. S3). Form IA genes were targeted with one primer pair because other primer pairs tested were not specific (resulting in multiple banding of PCR products).

Autotrophy in *Thaumarchaeota* was investigated by the analysis of genes coding for 4-hydroxybutyryl-CoA dehydratase (*hcd*), a key protein in the HP/HB and dicarboxylate/4-hydroxybutyrate (DC/4-HB) cycle (Berg, 2011). Several PCR based protocols were developed, and we selected two primers sets that produced specific amplification products in our samples (Offre *et al.* 2010; Yakimov *et al.* 2011, Table S1). For the quantification of *hcd* genes, a real time PCR primer pair (qPCR_hcd_f/qPCR_hcd_r, Table 1) was designed that specifically covers the sequence diversity of hcd genes obtained in this study. This primer was specifically designed on basis of the *hcd* sequences derived from PCR products with primers hcd-465F/hcd-1267R and 4HBD312F/4HBD1360R (Table

S1) of lakes ACH, STA and ZUR (Table S1, Fig. 2), and sequences of representatives within the marine Group I (MGI) *Thaumarchaeota* lineage. Gel electrophoresis and sequence analysis of selected PCR-amplificates produced with the qPCR primer from different samples of lakes ACH, STA and ZUR support the appropriateness of the qPCR assay (Fig. S3 and S4).

CO_2 fixation based on the rTCA cycle is generally detected by targeting genes coding for the alpha or beta subunit of the ATP citrate lyase (*aclAB* genes), and/or the alpha subunit of 2-oxoglutarate:ferredoxin oxidoreductase enzymes (*oorA* genes). Published primer sets, which were tested on samples from our study sites, mostly produced multiple bands or faint amplification products. Furthermore, sequence analysis of randomly selected PCR amplicons of *aclAB* genes produced nucleotide sequences that were mostly not affiliated with the target genes (data not shown). In fact, most published primers were originally designed to study marine hydrothermal environments; therefore, these primers specifically target thermophilic *Aquificales* and *Epsilonproteobacteria* (reviewed by Hügler and Sievert, 2011). Further primer optimization was necessary and we developed a broad range detection system, based on a nested PCR approach, targeting genes coding for ATP citrate lyase alpha subunit (*aclA*) within different clades of *Epsilonproteobacteria*, *Deltaproteobacteria*, *Acidobacteria*, and *Nitrospirae*. A second primer set was developed to specifically target *aclA* genes in *Nitrospirae*. Finally, eleven primer systems were used to amplify different functional genes coding for key enzymes in the Calvin cycle, the HP/HB cycle, and the rTCA cycle in samples from six stratified lakes (Table S1).

Flow cytomtry

Total prokaryotic abundances and numbers of photoautotrophic cells in lakes EGE and ZUR were quantified by an inFlux V-GS cell sorter (Becton Dickinson, Franklin Lakes, NJ, USA) equipped with a ultraviolet (UV) laser (Lightwave Electronics, CY-PS, 60 mW, wavelength of 355 nm), a blue laser (Coherent, Saphire, 200 mW, wavelength of 488 nm), and detectors for two scatter and six fluorescence channels. All samples were stained with 4,6-diamidino-2-phenylindole (DAPI, 1 mg ml-1 final concentration), and scatter plots were analyzed with an in-house software (J Villiger, unpublished).

Table S1. Specification of primers applied for the detection of different CO_2 fixation pathways

Pathway, Target gene	Primer Name	Sequence (5´-3´)	bp	Ta[1]	Reference
**CBB-cycle**					
RubisCO form IA	cbbL_IA_f	CGGCACSTGGACCACSGTSTGGAC	620	54	Alfreider *et al.* (2003)
	cbbL_IA_r	GTARTCGTGCATGATGATSGG			
RubisCO form IC	cbbL_IC_f	GAACATCAAYTCKCAGCCCTT	552	55	Alfreider *et al.* (2009)
	cbbL_IC_r	TGGTGCATCTGVCCGGCRTG			
	cbbLR1F	AAGGAYGACGAGAACATC	818	57	Selesi *et al.* (2009)
	cbbLR1R	TCGGTCGGSGTGTAGTTGAA			
RubisCO form II	cbbM_f	GGCACCATCATCAAGCCCAAG	505	53	Alfreider *et al.* (2003)
	cbbM_r	TCTTGCCGTAGCCCATGGTGC			
	cbbM343F	GGYAAYAACCARGGYATGGG	783	50	Kato *et al.* (2012)
	cbbM1226R	CGYARBGCRTTCATRCCRCC			
**HP/HB cycle**					
4-hydroxybutyryl- CoA dehydratase	hcd-465F	GGHGGTGCWATGACTGA	839	53	Offre *et al.* (2010)
	hcd-1267R	CTCATTCTGTTTTCHACATC			
	4HBD 312F	TTCCAAAGATGTGTYGGWATGG	1048	50	Yakimov *et al.* (2011)
	4HBD 1360R	CCATGCATTGATTCHGTAA			
Quantitative PCR	qPCR_hcd_f	GACTGATCCWAAAGGDGAYAGAAG	227	56	This study
	qPCR_hcd_r	CCYTTARCATCTGCWGGAATTGC			
Acetyl-CoA/propionyl- CoA carboxylase	accA_f	CCNGCHATGACNGAYTTTG	481	49	This study
	accA_r	ATRTCWARYGCRCCWGCAAG			
**rTCA pathway**					
ATP citrate lyase (broad range)	aclA_f680	TNGGHGARRTNGGNGG	811	50	This study (nested PCR, primer set 1)[2]
	aclA_r1491	AYDCKRTGNCCDATNCC			
	aclA_f807	TDMARTTYGGNCAYGCNGGNGC	564	55	This study (nested PCR, primer set 2)[2]
	aclA_r1371	GCNCCDCCRAANCKNGGNCCDAT			
ATP-citrate lyase (*Nitrospira*)	aclA_Nitro_f	GGCATGAAGGGTGAAGGATC	714	52	This study
	aclA_Nitro_r	AACTCTTCACGTACCCGACC			

[1] Ta: Annealing temperature; [2] Details for nested PCR see Material and Method section

Table S2. Summary of physico-chemical parameters in the studied lakes

Depth (m)	Temp* °C	pH	Cond** µS cm^{-1}	O_2 mg L^{-1}	Ptot$^+$ µg L^{-1}	NH_4^+ µg L^{-1}	NO_3^- µg L^{-1}	SO_4^{2-} mg L^{-1}	DOC µg L-1	HCO_3^- mmol L^{-1}
**ZUR**										
20	7.4	7.58	296.0	4.8	10.0	5	784	14.90	1250	2.62
60	4.4	7.76	293.0	8.4	15.0	b.d.	764	15.00	n.d.	2.53
80	4.2	7.73	295.0	7.2	26.0	b.d.	764	15.10	n.d.	2.57
100	4.2	7.68	297.0	5.8	38.0	3	777	15.10	n.d.	2.60
115	4.2	7.58	301.0	2.8	50.0	3	797	14.80	n.d.	2.62
120	4.2	7.49	302.0	1.0	99.0	165	443	14.80	n.d.	2.66
130	4.2	7.47	306.0	0.1	152.0	460	210	14.10	n.d.	2.74
135	4.2	7.45	307.0	0.1	155.0	562	192	13.70	n.d.	2.75
**ACH**										
0	10.2	8.37	274	9.6	1.8	1	406	2.88	1136	2.82
10	10.4	8.42	274	9.8	2.4	1	407	2.88	1136	2.83
20	9.7	8.39	277	10.1	2.4	1	408	2.88	1251	2.83
25	7.5	8.32	277	10.4	2.1	1	460	2.98	1149	2.82
30	5.9	8.29	278	10.4	1.8	1	484	3.00	1428	2.83
60	5.0	8.29	279	10.5	1.5	1	478	3.07	1039	2.86
100	5.0	8.23	282	9.9	1.5	1	477	3.12	1011	2.88
129	4.9	8.19	284	9.8	2.4	5	467	3.16	1027	2.90
**STA**										
1	20.7	8.50	292	8.9	3.8	5	206	8.18	405	2.60
9	18.3	8.58	295	8.9	5.5	5	217	8.22	399	2.60
10	15.5	8.62	293	9.3	4.1	5	207	8.22	399	2.59
13	10.9	8.26	317	9.7	6.1	1	382	8.40	512	2.81
19	6.9	8.09	321	9.1	6.3	7	451	8.55	547	2.81
30	4.8	8.06	322	9.5	4.5	1	462	8.64	552	2.81
80	4.2	8.09	320	10.1	3.6	3	446	8.63	553	2.82
100	4.2	8.07	322	9.6	4.2	2	442	8.59	538	2.84
**HEC**										
0	25.4	8.62	310	9.3	7.4	5	254	4.32	3659	3.02
5	15.7	8.55	350	13.1	12.0	7	358	5.24	3628	3.43
10	7.2	8.17	366	8.3	8.8	28	445	5.88	2994	3.54
12	6.1	n.d	n.d.	3.4	n.d	n.d	n.d	n.d	n.d.	n.d
14	5.7	n.d	n.d	0.3	n.d	n.d	n.d	n.d	n.d.	n.d
15	5.7	7.83	388	0.4	11.4	29	459	6.50	2752	3.76
25	6.3	7.39	539	b.d.	167.5	4168	7	4.02	2176	5.54
50	6.7	7.34	578	b.d.	320.7	5638	18	2.56	2434	5.97
**PIB**										
0	17.2	7.68	67	8.8	4.4	15	184	5.78	2382	0.463
3	16.9	7.79	67	8.8	5.6	15	184	5.75	2368	0.462
6	12.2	7.50	70	10.4	10.9	10	168	6.14	2194	0.493
9	7.2	7.01	73	7.2	7.6	7	220	6.38	1870	0.510
12	5.6	6.84	73	4.5	8.8	15	251	6.34	1754	0.512
15	4.9	6.71	74	1.6	7.1	60	251	6.29	1724	0.522
18	4.8	6.66	75	0.2	11.8	120	167	6.09	1814	0.540
21	4.6	6.65	77	0.1	12.6	240	54	5.99	1823	0.566
24	4.6	6.65	79	0.0	19.7	415	8	5.66	1949	0.586
**EGE**										
5	15.7	n.d.	393	7.01	16.5	4	7	68.8	3524	4.21
5.5	14.3	n.d	418	5.94	33.5	6	5	53.9	3747	4.53
5.75	13.4	n.d	421	4.21	45.8	4	7	49.4	3788	4.63
6	12.8	n.d	428	2.04	64.3	5	7	46.6	3744	4.69
6.25	11.4	n.d	437	0.93	89.2	6	11	48.0	3753	4.73
6.5	10.8	n.d	441	0.66	133.3	261	10	37.0	4014	4.745
6.75	10.0	n.d	448	0.47	89.7	788	0	22.7	3461	4.87
7	9.3	n.d	453	0.36	82.0	1297	0	16.5	3497	4.97
8.5	6.5	n.d	505	b.d.	79.0	3840	0	15.4	5516	5.12
10	5.7	n.d	593	b.d.	152.5	8532	0	15.8	6590	5.52

* Temperature
** Conductivity

Fig. S1. Neighbor-Joining trees obtained from deduced amino acid sequences of forms IA (Fig. S1A), IC (Fig. S1B) and II (Fig. S1C) RubisCO genes in the CBB cycle derived from this study (shown in blue letters) and representative sequences from NCBI and IMG databases. Bootstrap values are shown as percentages of 1000 replicates and values over 50% are indicated on nodes. Scale bar indicates 5 % changes in the amino acid sequence. Vertical bars with different colors, as identified in the legend inset, indicate phylogenetic affiliation of closest relatives. Where applicable, potential functional traits derived from physiological properties of the cultured closest relatives are designated by differently colored dots.

Fig. S1A

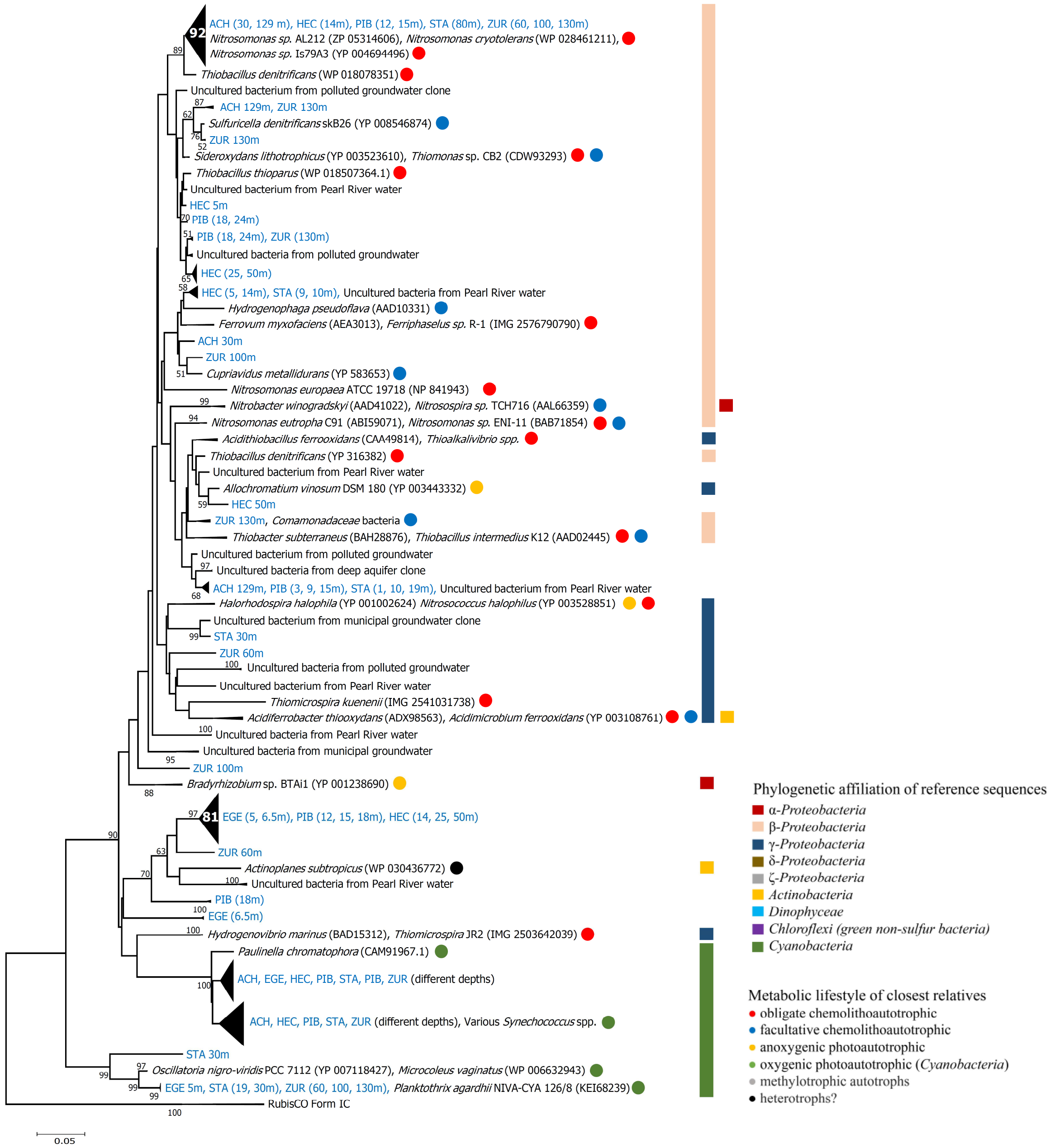

Fig. S1B

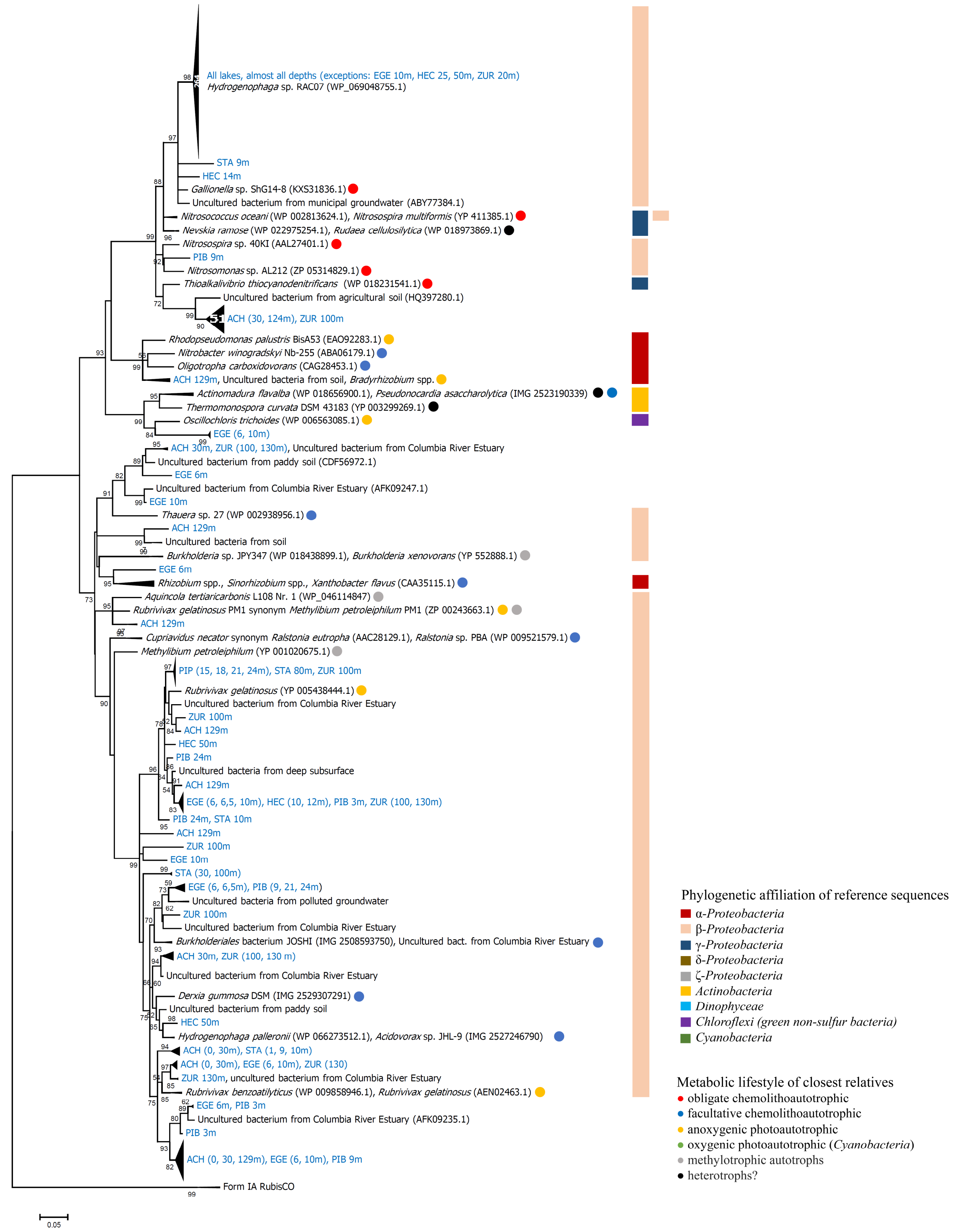

All lakes, almost all depths (exceptions: EGE 10m, HEC 25, 50m, ZUR 20m)
Hydrogenophaga sp. RAC07 (WP_069048755.1)
STA 9m
HEC 14m
Gallionella sp. ShG14-8 (KXS31836.1)
Uncultured bacterium from municipal groundwater (ABY77384.1)
Nitrosococcus oceani (WP 002813624.1), Nitrosospira multiformis (YP 411385.1)
Nevskia ramose (WP 022975254.1), Rudaea cellulosilytica (WP 018973869.1)
Nitrosospira sp. 40KI (AAL27401.1)
PIB 9m
Nitrosomonas sp. AL212 (ZP 05314829.1)
Thioalkalivibrio thiocyanodenitrificans (WP 018231541.1)
Uncultured bacterium from agricultural soil (HQ397280.1)
ACH (30, 124m), ZUR 100m
Rhodopseudomonas palustris BisA53 (EAO92283.1)
Nitrobacter winogradskyi Nb-255 (ABA06179.1)
Oligotropha carboxidovorans (CAG28453.1)
ACH 129m, Uncultured bacteria from soil, Bradyrhizobium spp.
Actinomadura flavalba (WP 018656900.1), Pseudonocardia asaccharolytica (IMG 2523190339)
Thermomonospora curvata DSM 43183 (YP 003299269.1)
Oscillochloris trichoides (WP 006563085.1)
EGE (6, 10m)
ACH 30m, ZUR (100, 130m), Uncultured bacterium from Columbia River Estuary
Uncultured bacterium from paddy soil (CDF56972.1)
EGE 6m
Uncultured bacterium from Columbia River Estuary (AFK09247.1)
EGE 10m
Thauera sp. 27 (WP 002938956.1)
ACH 129m
Uncultured bacteria from soil
Burkholderia sp. JPY347 (WP 018438899.1), Burkholderia xenovorans (YP 552888.1)
EGE 6m
Rhizobium spp., Sinorhizobium spp., Xanthobacter flavus (CAA35115.1)
Aquincola tertiaricarbonis L108 Nr. 1 (WP_046114847)
Rubrivivax gelatinosus PM1 synonym Methylibium petroleiphilum PM1 (ZP 00243663.1)
ACH 129m
Cupriavidus necator synonym Ralstonia eutropha (AAC28129.1), Ralstonia sp. PBA (WP 009521579.1)
Methylibium petroleiphilum (YP 001020675.1)
PIP (15, 18, 21, 24m), STA 80m, ZUR 100m
Rubrivivax gelatinosus (YP 005438444.1)
Uncultured bacterium from Columbia River Estuary
ZUR 100m
ACH 129m
HEC 50m
PIB 24m
Uncultured bacteria from deep subsurface
ACH 129m
EGE (6, 6,5, 10m), HEC (10, 12m), PIB 3m, ZUR (100, 130m)
PIB 24m, STA 10m
ACH 129m
ZUR 100m
EGE 10m
STA (30, 100m)
EGE (6, 6,5m), PIB (9, 21, 24m)
Uncultured bacteria from polluted groundwater
ZUR 100m
Uncultured bacterium from Columbia River Estuary
Burkholderiales bacterium JOSHI (IMG 2508593750), Uncultured bact. from Columbia River Estuary
ACH 30m, ZUR (100, 130 m)
Uncultured bacterium from Columbia River Estuary
Derxia gummosa DSM (IMG 2529307291)
Uncultured bacterium from paddy soil
HEC 50m
Hydrogenophaga palleronii (WP 066273512.1), Acidovorax sp. JHL-9 (IMG 2527246790)
ACH (0, 30m), STA (1, 9, 10m)
ACH (0, 30m), EGE (6, 10m), ZUR (130)
ZUR 130m, uncultured bacterium from Columbia River Estuary
Rubrivivax benzoatilyticus (WP 009858946.1), Rubrivivax gelatinosus (AEN02463.1)
EGE 6m, PIB 3m
Uncultured bacterium from Columbia River Estuary (AFK09235.1)
PIB 3m
ACH (0, 30, 129m), EGE (6, 10m), PIB 9m
Form IA RubisCO
0.05
Phylogenetic affiliation of reference sequences
α-Proteobacteria
β-Proteobacteria
γ-Proteobacteria
δ-Proteobacteria
ζ-Proteobacteria
Actinobacteria
Dinophyceae
Chloroflexi (green non-sulfur bacteria)
Cyanobacteria
Metabolic lifestyle of closest relatives
obligate chemolithoautotrophic
facultative chemolithoautotrophic
anoxygenic photoautotrophic
oxygenic photoautotrophic (Cyanobacteria)
methylotrophic autotrophs
heterotrophs?

Fig. S1C

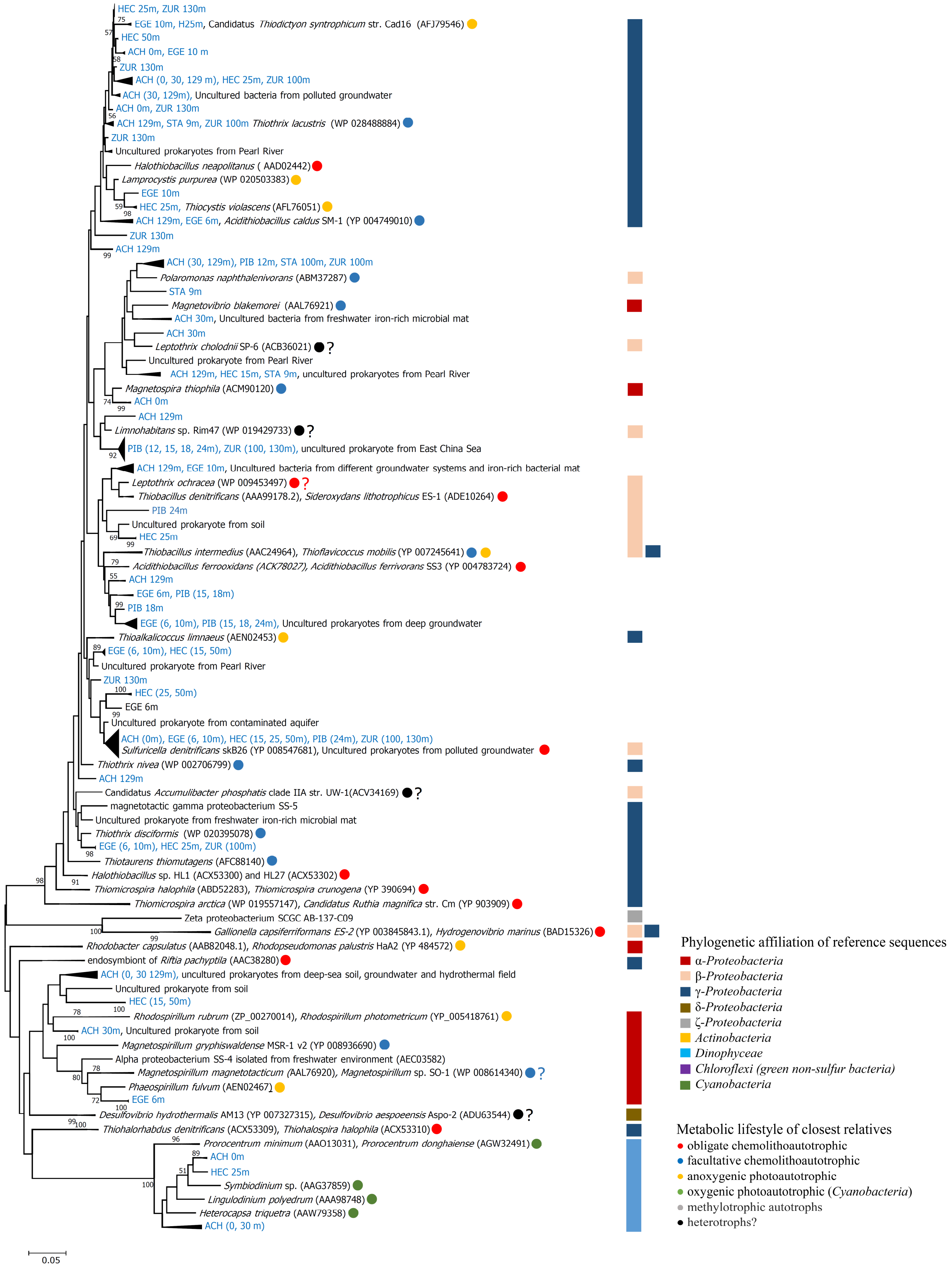
HEC 25m, ZUR 130m
EGE 10m, H25m, Candidatus Thiodictyon syntrophicum str. Cad16 (AFJ79546)
HEC 50m
ACH 0m, EGE 10 m
ZUR 130m
ACH (0, 30, 129 m), HEC 25m, ZUR 100m
ACH (30, 129m), Uncultured bacteria from polluted groundwater
ACH 0m, ZUR 130m
ACH 129m, STA 9m, ZUR 100m Thiothrix lacustris (WP 028488884)
ZUR 130m
Uncultured prokaryotes from Pearl River
Halothiobacillus neapolitanus (AAD02442)
Lamprocystis purpurea (WP 020503383)
EGE 10m
HEC 25m, Thiocystis violascens (AFL76051)
ACH 129m, EGE 6m, Acidithiobacillus caldus SM-1 (YP 004749010)
ZUR 130m
ACH 129m
ACH (30, 129m), PIB 12m, STA 100m, ZUR 100m
Polaromonas naphthalenivorans (ABM37287)
STA 9m
Magnetovibrio blakemorei (AAL76921)
ACH 30m, Uncultured bacteria from freshwater iron-rich microbial mat
ACH 30m
Leptothrix cholodnii SP-6 (ACB36021) ?
Uncultured prokaryote from Pearl River
ACH 129m, HEC 15m, STA 9m, uncultured prokaryotes from Pearl River
Magnetospira thiophila (ACM90120)
ACH 0m
ACH 129m
Limnohabitans sp. Rim47 (WP 019429733) ?
PIB (12, 15, 18, 24m), ZUR (100, 130m), uncultured prokaryote from East China Sea
ACH 129m, EGE 10m, Uncultured bacteria from different groundwater systems and iron-rich bacterial mat
Leptothrix ochracea (WP 009453497) ?
Thiobacillus denitrificans (AAA99178.2), Sideroxydans lithotrophicus ES-1 (ADE10264)
PIB 24m
Uncultured prokaryote from soil
HEC 25m
Thiobacillus intermedius (AAC24964), Thioflavicoccus mobilis (YP 007245641)
Acidithiobacillus ferrooxidans (ACK78027), Acidithiobacillus ferrivorans SS3 (YP 004783724)
ACH 129m
EGE 6m, PIB (15, 18m)
PIB 18m
EGE (6, 10m), PIB (15, 18, 24m), Uncultured prokaryotes from deep groundwater
Thioalkalicoccus limnaeus (AEN02453)
EGE (6, 10m), HEC (15, 50m)
Uncultured prokaryote from Pearl River
ZUR 130m
HEC (25, 50m)
EGE 6m
Uncultured prokaryote from contaminated aquifer
ACH (0m), EGE (6, 10m), HEC (15, 25, 50m), PIB (24m), ZUR (100, 130m)
Sulfuricella denitrificans skB26 (YP 008547681), Uncultured prokaryotes from polluted groundwater
Thiothrix nivea (WP 002706799)
ACH 129m
Candidatus Accumulibacter phosphatis clade IIA str. UW-1(ACV34169) ?
magnetotactic gamma proteobacterium SS-5
Uncultured prokaryote from freshwater iron-rich microbial mat
Thiothrix disciformis (WP 020395078)
EGE (6, 10m), HEC 25m, ZUR (100m)
Thiotaurens thiomutagens (AFC88140)
Halothiobacillus sp. HL1 (ACX53300) and HL27 (ACX53302)
Thiomicrospira halophila (ABD52283), Thiomicrospira crunogena (YP 390694)
Thiomicrospira arctica (WP 019557147), Candidatus Ruthia magnifica str. Cm (YP 903909)
Zeta proteobacterium SCGC AB-137-C09
Gallionella capsiferriformans ES-2 (YP 003845843.1), Hydrogenovibrio marinus (BAD15326)
Rhodobacter capsulatus (AAB82048.1), Rhodopseudomonas palustris HaA2 (YP 484572)
endosymbiont of Riftia pachyptila (AAC38280)
ACH (0, 30 129m), uncultured prokaryotes from deep-sea soil, groundwater and hydrothermal field
Uncultured prokaryote from soil
HEC (15, 50m)
Rhodospirillum rubrum (ZP_00270014), Rhodospirillum photometricum (YP_005418761)
ACH 30m, Uncultured prokaryote from soil
Magnetospirillum gryphiswaldense MSR-1 v2 (YP 008936690)
Alpha proteobacterium SS-4 isolated from freshwater environment (AEC03582)
Magnetospirillum magnetotacticum (AAL76920), Magnetospirillum sp. SO-1 (WP 008614340) ?
Phaeospirillum fulvum (AEN02467)
EGE 6m
Desulfovibrio hydrothermalis AM13 (YP 007327315), Desulfovibrio aespoeensis Aspo-2 (ADU63544) ?
Thiohalorhabdus denitrificans (ACX53309), Thiohalospira halophila (ACX53310)
Prorocentrum minimum (AAO13031), Prorocentrum donghaiense (AGW32491)
ACH 0m
HEC 25m
Symbiodinium sp. (AAG37859)
Lingulodinium polyedrum (AAA98748)
Heterocapsa triquetra (AAW79358)
ACH (0, 30 m)
0.05
Phylogenetic affiliation of reference sequences
α-Proteobacteria
β-Proteobacteria
γ-Proteobacteria
δ-Proteobacteria
ζ-Proteobacteria
Actinobacteria
Dinophyceae
Chloroflexi (green non-sulfur bacteria)
Cyanobacteria
Metabolic lifestyle of closest relatives
obligate chemolithoautotrophic
facultative chemolithoautotrophic
anoxygenic photoautotrophic
oxygenic photoautotrophic (Cyanobacteria)
methylotrophic autotrophs
heterotrophs?

Fig. S2. Coverage of two different primer sets targeting Form II RubisCO sequences (see Table S1) retrieved from different samples of the studied lakes. The green color indicates *cbbM* lineages that are covered by both primer pairs (Alfreider *et al*., 2003 and Kato *et al*., 2012).

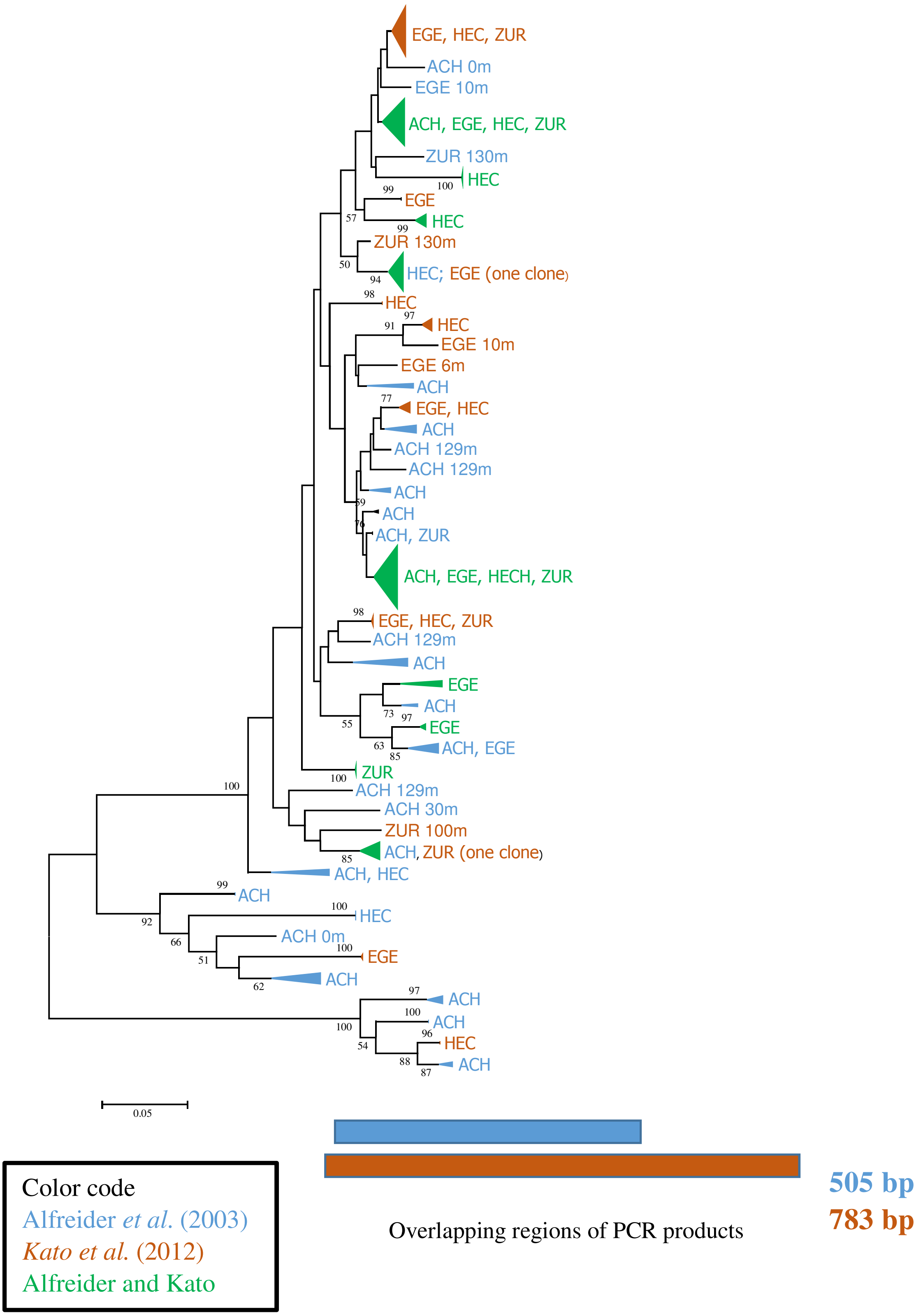

Fig. S3. Coverage of two different primer sets targeting Form 1C RubisCO sequences (see Table S1) retrieved from different samples of the studied lakes. The green color indicates *cbbL* IC lineages that are covered by both primer pairs (Alfreider *et al*., 2009 and Selesi *et al*., 2009).

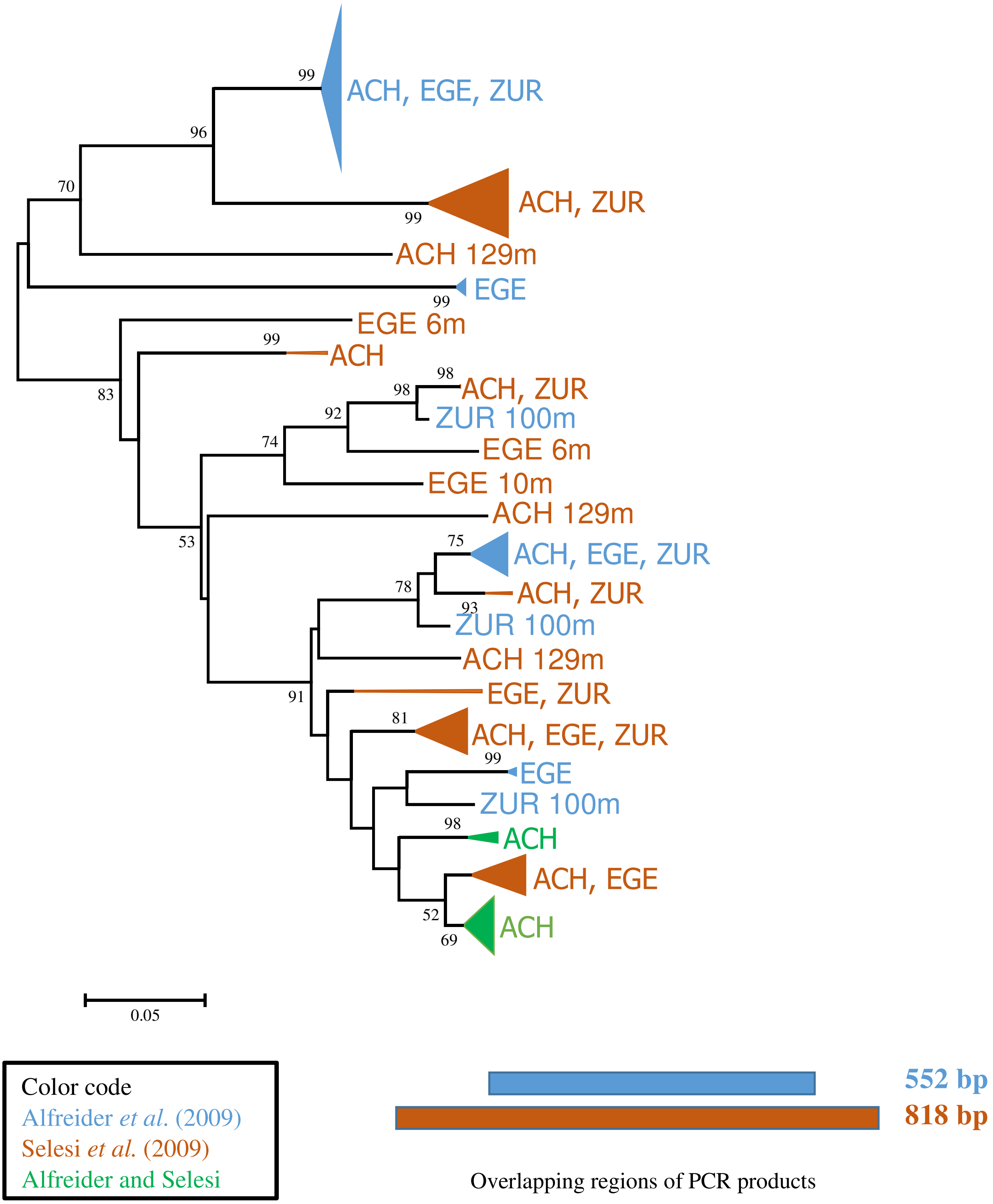

Fig. S4. Evaluation of different annealing temperatures for thaumarchaeal *hcd* qPCR primers with different samples of lakes ACH (0m, 129m), STA (30m) and ZUR (130). A sample from lake PIB (18m) was used as negative control.

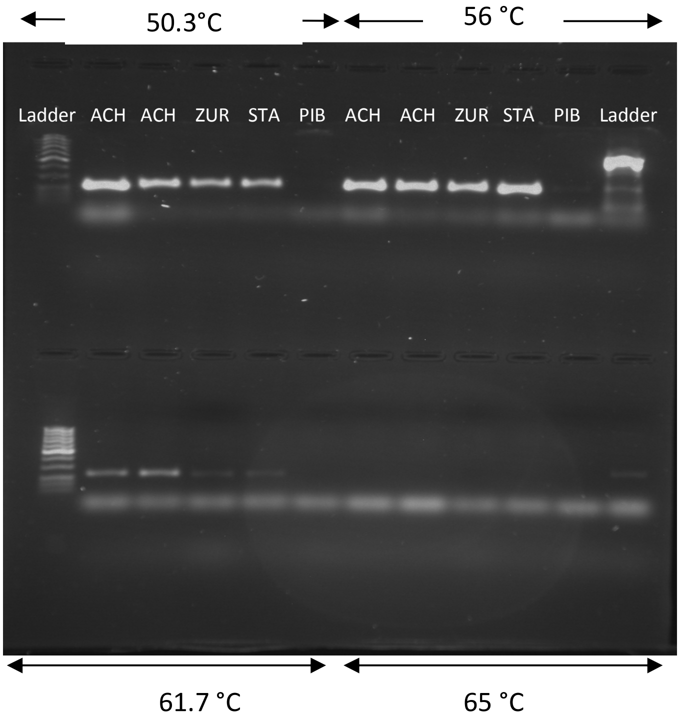

Fig. S5. Evaluation of the specificity of *hcd* qPCR primers (annealing temperature 56°C) by sequencing of cloned PCR products derived from samples of lakes ACH, STA and ZUR.

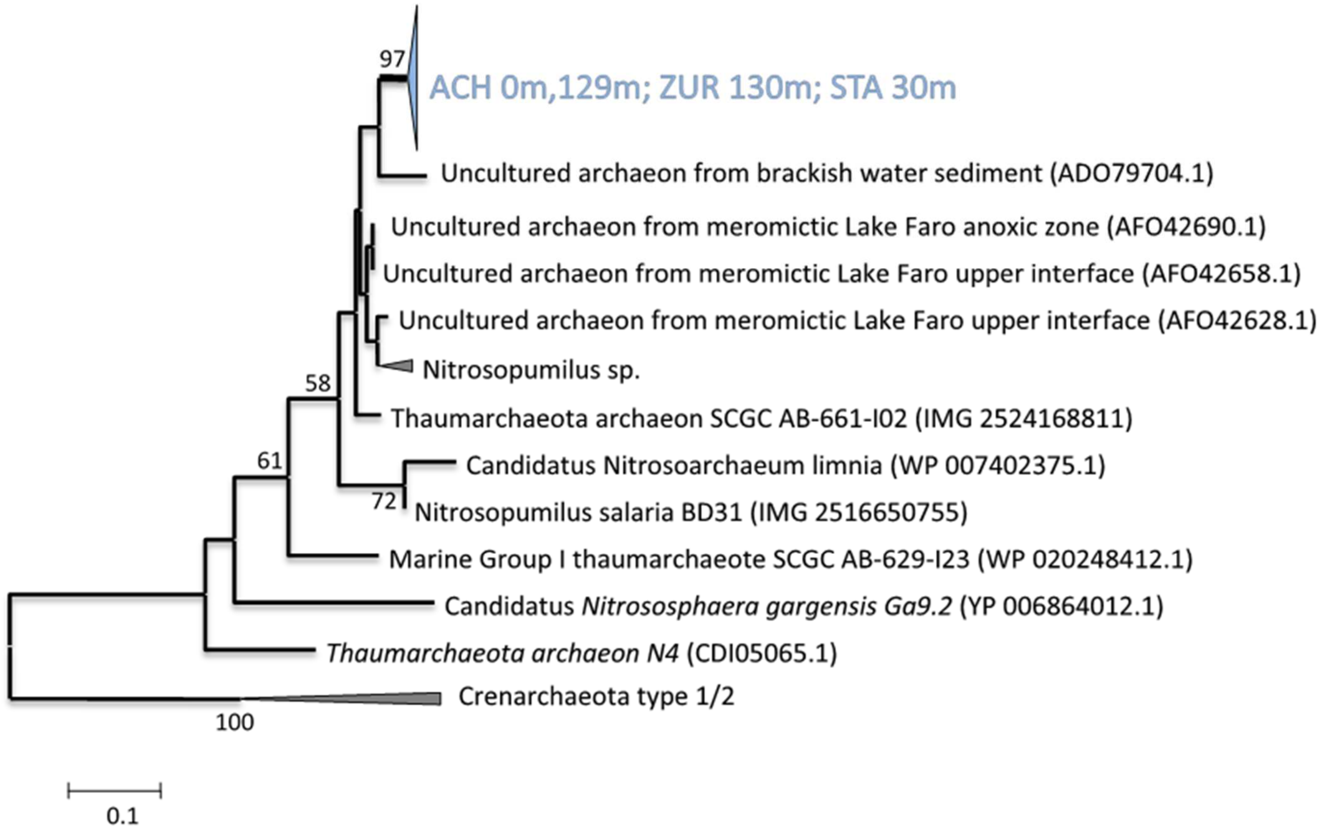

Fig. S6. Vertical profiles of ammonium, nitrate and oxygen in the studied lakes at the time of sampling.

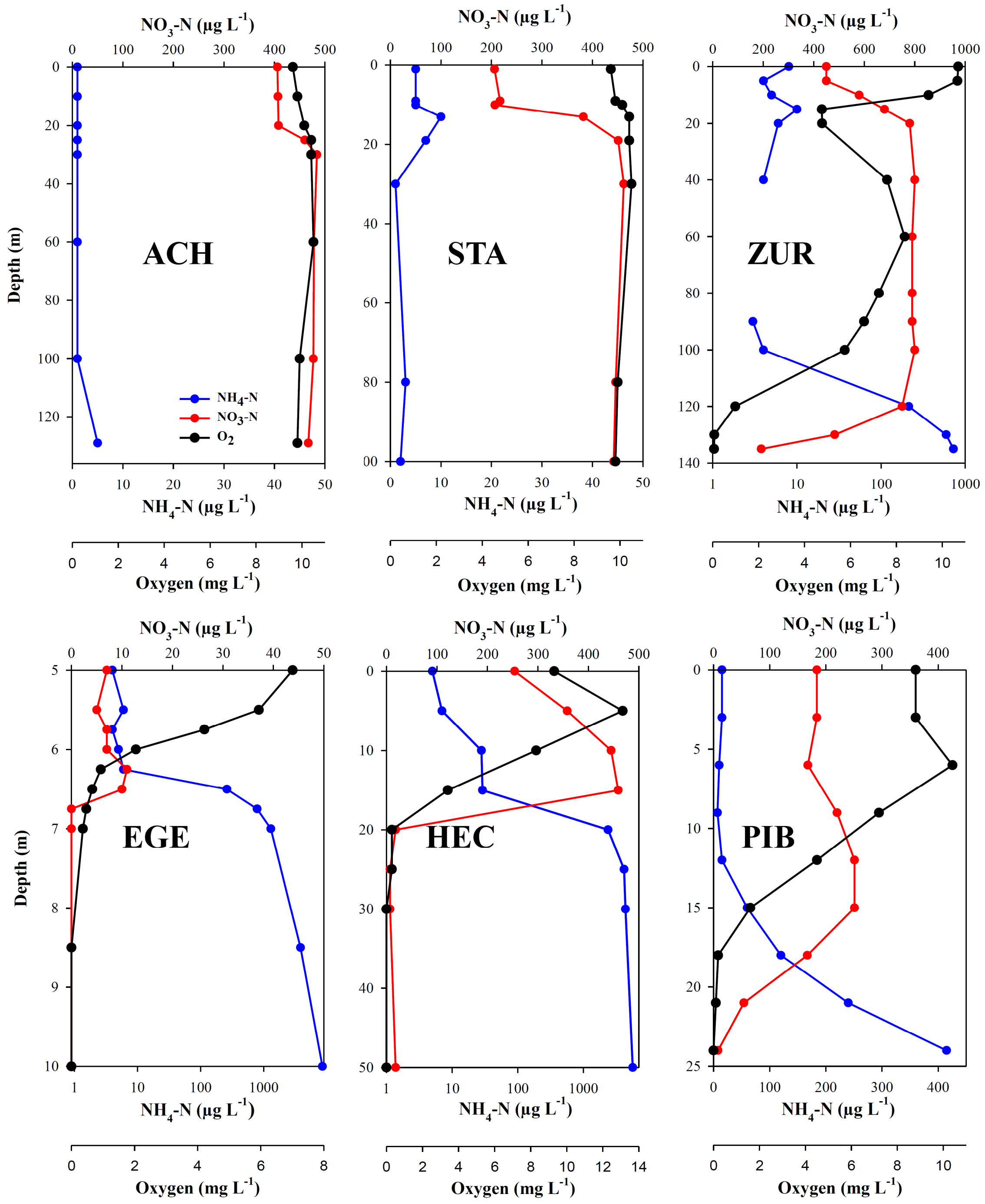

Fig. S7. Vertical profile of photoautotrophic cell numbers in Lake EGE

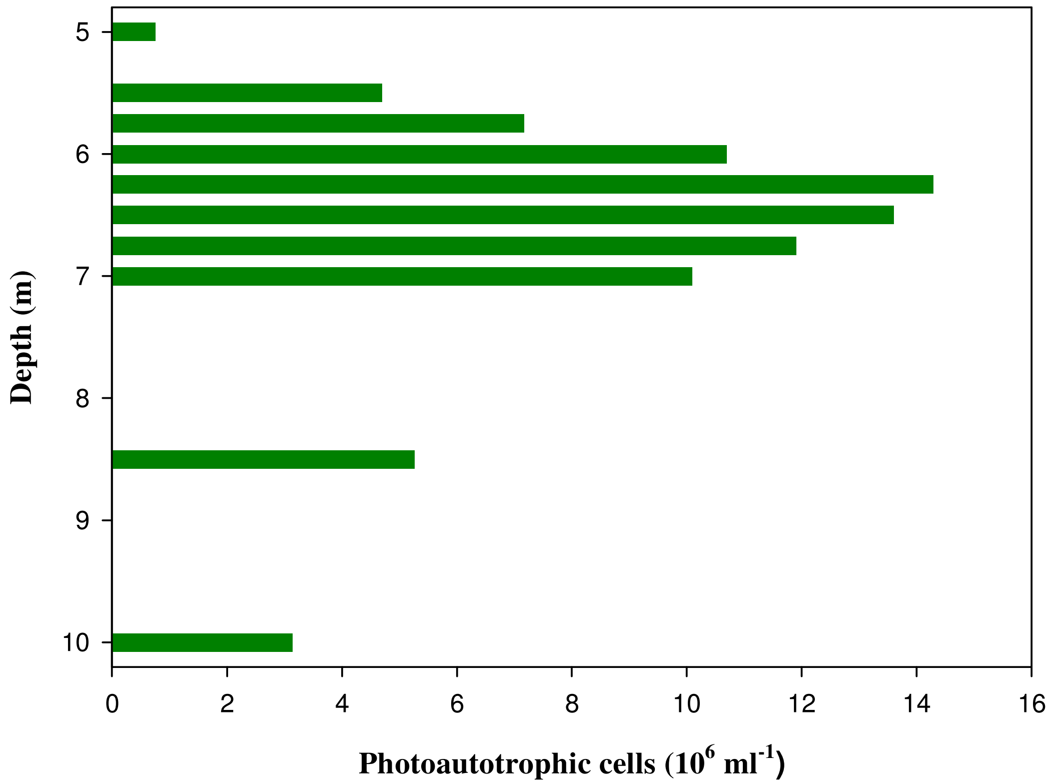

Supplementary References (not included in main text)

Alfreider, A., Vogt, C., Hoffmann, D., and Babel, W. (2003) Diversity of ribulose-1,5-bisphosphate carboxylase/oxygenase large-subunit genes from groundwater and aquifer microorganisms. *Microb Ecol* 45: 317-328.

Offre, P.R., Nicol, G.W., and Prosser, J.I. (2010) Autotrophic community profiling and quantification of putative autotrophic thaumarchaeal communities in environmental samples. E*nviron Microbiol Reports* 3: 245-253.

Kato, S., Nakawake, M., Ohkuma, M., and Yamagishi, A. (2012) Distribution and phylogenetic diversity of *cbbM* genes encoding RubisCO form II in a deep-sea hydrothermal field revealed by newly designed PCR primers. *Extremophiles* 16: 277-283.

Selesi. D., Schmid, M., and Hartmann, A. (2005) Diversity of green-like and red-like ribulose-1,5-bisphosphate carboxylase/oxygenase large-subunit genes (*cbbL*) in differently managed agricultural soils. *Appl Environ Microbiol* 77: 175–184
